# Identify adolescents' help-seeking intention on suicide through self- and caregiver's assessments of psychobehavioral problems: deep clustering of the Tokyo TEEN Cohort study

**DOI:** 10.1016/j.lanwpc.2023.100979

**Published:** 2023-12-13

**Authors:** Daiki Nagaoka, Akito Uno, Satoshi Usami, Riki Tanaka, Rin Minami, Yutaka Sawai, Ayako Okuma, Syudo Yamasaki, Mitsuhiro Miyashita, Atsushi Nishida, Kiyoto Kasai, Shuntaro Ando

**Affiliations:** a The Department of Neuropsychiatry, The University of Tokyo, Tokyo, Japan; b The Graduate School of Education, The University of Tokyo, Tokyo, Japan; c Research Center for Social Science & Medicine, Tokyo Metropolitan Institute of Medical Science, Tokyo, Japan; d The International Research Center for Neurointelligence (WPI-IRCN) at the University of Tokyo Institutes for Advanced Study (UTIAS), Tokyo, Japan

**Keywords:** Adolescent, Psychopathology, Comorbidity, Longitudinal, Trajectory, Cohort study, Deep learning, Clustering, Psychopathological and behavioral problems, Psychobehavioral problems

## Abstract

**Background:**

Psychopathological and behavioral problems in adolescence are highly comorbid, making their developmental trajectories complex and unclear partly due to technical limitations. We aimed to classify these trajectories using deep learning and identify predictors of cluster membership.

**Methods:**

We conducted a population-based cohort study on 3171 adolescents from three Tokyo municipalities, with 2344 pairs of adolescents and caregivers participating at all four timepoints (ages 10, 12, 14, and 16) from 2012 to 2021. Adolescent psychopathological and behavioral problems were assessed by using self-report questionnaires. Both adolescents and caregivers assessed depression/anxiety and psychotic-like experiences. Caregivers assessed obsession/compulsion, dissociation, sociality problem, hyperactivity/inattention, conduct problem, somatic symptom, and withdrawal. Adolescents assessed desire for slimness, self-harm, and suicidal ideation. These trajectories were clustered with variational deep embedding with recurrence, and predictors were explored using multinomial logistic regression.

**Findings:**

Five clusters were identified: *unaffected* (60.5%), minimal problems; *internalizing* (16.2%), persistent or worsening internalizing problems; *discrepant* (9.9%), subjective problems overlooked by caregivers; *externalizing* (9.6%), persistent externalizing problems; and *severe* (3.9%), chronic severe problems across symptoms. Stronger autistic traits and experience of bullying victimization commonly predicted the four “affected” clusters. The *discrepant* cluster, showing the highest risks for self-harm and suicidal ideation, was predicted by avoiding help-seeking for depression. The *severe* cluster predictors included maternal smoking during pregnancy, not bullying others, caregiver's psychological distress, and adolescent's dissatisfaction with family.

**Interpretation:**

Approximately 40% of adolescents were classified as “affected” clusters. Proactive societal attention is warranted toward adolescents in the *discrepant* cluster whose suicidality is overlooked and who have difficulty seeking help.

**Funding:**

Japan Ministry of Health, Labor and Welfare, Japan Agency for Medical Research and Development, and 10.13039/501100002241Japan Science and Technology Agency.

## Translated abstract

**背景:**

思春期の精神病理的問題や行動上の問題は併存しやすい。これらの問題が時間的に発展する軌跡は複雑であり、一部技術的な制限のために不明な点も多く残っている。 本研究の目的は、深層学習に基づくクラスタリングによりこれらの軌跡を分類することと、そのクラスタへの所属を予測する因子を同定することとした。

**方法:**

東京都の3つの自治体（世田谷区、三鷹市、調布市）から無作為に抽出した3171名の思春期児童を対象に、一般集団ベースの前向きコホート研究を実施した。このうち、2012年から2021年までの4つの時点（10歳、12歳、14歳、16歳）すべてに参加した思春期児童とその養育者2344組を対象とした。それぞれ自己報告式の質問票を用いて思春期児童の精神病理的および行動上の問題を評価した。抑うつ/不安と精神病様体験は、思春期児童と養育者の両方によって評価された。強迫観念/強迫行為、解離、社会性の問題、多動/不注意、行動の問題、身体症状、引きこもりは養育者が評価した。痩せ願望、自傷行為、希死念慮は思春期児童が評価した。これらの軌跡のクラスタリングを再帰的変分深層埋め込み法（variational deep embedding with recurrence）で行い、多項ロジスティック回帰で予測因子の探索を行った。

**結果:**

以下の5つのクラスタが同定された：問題が最小限であった「非影響群」（60.5％）、持続的または悪化する内在化問題を示した「内在化群」（16.2％）、児童の主観的な問題が養育者に見過ごされた「乖離群」（9.9％）、持続的な外在化問題を示した「外在化群」（9.6％）、症状全体にわたる慢性的な重度の問題を呈した「重度群」（3.9％）。4つの 「問題を呈するクラスタ」に共通する予測因子は、より強い自閉的特性といじめの被害経験だった。自傷行為と希死念慮のリスクが最も高い「乖離群」は、うつのとき助けを求めようとしないことによって予測された。「重度群」の予測因子には、母の妊娠中の喫煙、他人をいじめていないこと、養育者の心理的苦痛、思春期児童の家族に対する不満などがあった。

**解釈:**

約40％の思春期児童がいずれかの「問題を呈するクラスタ」に分類された。主観的な精神病理的問題が養育者に見過ごされていた「乖離群」は、自傷行為と希死念慮を最も多く呈した。自殺傾向が見過ごされ、助けを求めることが困難な思春期児童に対する社会全般からの積極的な注意が必要である。

**研究助成:**

厚生労働省、日本医療研究開発機構、科学技術振興機構.

**Disclaimer:**

This translation in Japanese was submitted by the authors and we reproduce it as supplied. It has not been peer reviewed. Our editorial processes have only been applied to the original abstract in English, which should serve as reference for this manuscript.

**Research in context**

**Evidence before this study**

We searched PubMed for articles published in English using the terms (“trajector∗” [Title] OR “develop∗” [Title]) AND (“adolescen∗” [Title/Abstract] OR “child∗” [Title/Abstract] OR “youth” [Title/Abstract]) AND (“mental” [Title/Abstract] OR “psychiatr∗” [Title/Abstract] OR “psychopatholog∗” [Title/Abstract]) AND (“emotion∗” [Title/Abstract] OR “internali∗” [Title/Abstract]) AND (“behavio∗” [Title/Abstract] OR “externali∗” [Title/Abstract] OR “conduct” [Title/Abstract]) AND (“comorbid∗” [Title/Abstract] OR “complicat∗” [Title/Abstract] OR “co-occur∗” [Title/Abstract] OR “cross∗” [Title/Abstract] OR “joint” [Title/Abstract]) AND (“cohort” [Title/Abstract] OR “general” [Title/Abstract]) before March 31, 2023. From the output of this search strategy, we selected studies that assess developmental trajectories of multiple psychopathological and behavioral symptoms in general adolescence. We also manually reviewed the reference lists of the selected publications. Two studies summarized the trajectories of internalizing and externalizing problems during childhood or adolescence as evaluated by parents into several latent classes, further investigating predictors of these class memberships. Another study incorporated symptoms of attention deficit/hyperactive disorder into the analysis, alongside internalizing and externalizing symptoms, as evaluated by teachers. These study reveals several patterns of psychopathological development, and predictors for the class exhibiting severe course, including male sex, younger maternal age, maternal smoking during pregnancy, conflict between parents and adolescents, maternal psychological distress, maternal psychiatric diagnosis, residence in poverty-stricken area, bullying victimization, low academic achievement, and polygenic risks. However, these still have limitations in understanding complex trajectories, such as applying mathematical models that require numerous assumptions, employing a questionnaire completed by a single evaluator without adolescent-self, and using simplified indices without considering the comorbidity of multiple symptoms over time.

**Added value of this study**

In the realm of research on the psychopathology of the general population of adolescents, our study introduces the value of incorporating evaluations from both the adolescents and their caregivers. This approach uncovered a *discrepant* cluster, wherein subjective distress has been overlooked by caregivers. Furthermore, we identified additional predictors for the cluster exhibiting severe psychopathological and behavioral trajectories across all problems: shorter weeks of gestation at delivery, caregiver-perceived difficulty in parenting at age three, poorer exercise habits, not bullying others, and adolescents' dissatisfaction with family.

**Implications of all the available evidence**

Adolescents demonstrating discrepancy in subjective and caregiver-objective assessments, and unable to seek help potentially exhibit elevated suicide-related risks. Therefore, proactive attention and support provision for these groups should be strongly considered.

## Introduction

The development of psychopathological and behavioral problems in adolescents is highly complex and heterogeneous.^1^ In adolescence, psychopathological symptoms are comorbid in diverse ways across various diagnostic categories based on adults. The comorbidities of psychopathological and behavioral problems are a crucial concern, as they are associated with more severe problems and increased suicide rates.^2^ To address the comorbidity, the International Classification of Diseases (ICD) and Diagnostic and Statistical Manual of Mental Disorders (DSM) are adopting a dimensional approach. Furthermore, the longitudinal trajectory of individual psychopathological symptoms is also diverse, including those that are transient, recurrent, and chronic.^3^ For example, the trajectories of psychopathological problems such as depressive symptoms and psychotic symptoms were divided into several groups, and each group had different characteristics and prognoses.^4^ However, most studies have focused on a single domain of psychopathology, despite the importance of capturing the comorbidity of multiple psychopathological and behavioral problems. Efforts to summarize the complex development of psychopathology, such as the p factor,^5^ transdiagnostic clinical staging,^6^ and the Hierarchical Taxonomy of Psychopathology (HiTOP),^7^ have offered valuable insights, including organization of co-occurring psychopathological symptoms and identification of common risk factors underlying their diverse manifestations. However, the p factor and HiTOP might be inadequate from a developmental perspective, as they primarily focus on cross-sectional symptom patterns. Likewise, the p factor and transdiagnostic clinical staging could also be insufficient for capturing the heterogeneity of symptom domains, as they emphasize commonalities across disorders. To fully understand the development of psychopathology, it is necessary to assess the trajectories of multiple symptoms together rather than focusing on limited symptoms.

Only recently have attempts been made to assess trajectories of multiple psychopathological and behavioral symptoms. Numerous studies have examined joint trajectories of various psychiatric symptoms in adolescence to identify latent classes, addressing up to three symptom domains concurrently.^8^ In the context of developmental psychopathology, several studies have summarized the trajectories of emotional (or internalizing) and behavioral (or externalizing) problems in the general population of children or adolescents into several latent classes and examined predictors of the class membership.^9^, ^10^, ^11^ Another study assessed the progression of internalizing and externalizing problems concurrently and discovered that adolescents may exhibit transitions between the four latent classes identified at each time point.^12^ However, these studies have limitations in understanding complex trajectories: applying mathematical models that require numerous assumptions such as specific trajectory shape and time-invariant latent classes, employing a questionnaire answered by a single evaluator, and using simplified indices without considering the comorbidity over time. To address these limitations, deep learning models can be useful, as they have demonstrated exceptional performance in learning latent representations of complex data. Such models can incorporate numerous assessments from multiple informants, thereby potentially enhancing our understanding of psychopathology.^13^ A model suitable for clustering longitudinal clinical or epidemiological data has also been proposed.^14^ By utilizing such models, we can better capture the comorbidity of multiple symptoms over time, providing a more comprehensive understanding of psychopathological and behavioral problems in adolescents.

This study has two primary objectives. First, we aim to cluster the general population of adolescents based on six-year trajectories of multiple psychopathological and behavioral problems using a deep learning model. Second, we compare demographic and environmental characteristics across the clusters and identify predictors for each cluster membership. By considering the comorbidity of symptoms longitudinally, we expect to contribute to a better understanding of psychopathological development in adolescence. Furthermore, incorporating multiple assessments by adolescents and caregivers into the model is also expected to provide a multidimensional view of the development of psychopathology.

## Methods

### Study design and survey participants

In this prospective population-based cohort study, the Tokyo Teen Cohort study (TTC),^15^ adolescents born between September 2002 and August 2004 were randomly selected from the Basic Resident Register of three municipalities in Tokyo (Appendix p 23). Data were collected at four time points from October 2012 to September 2021, when the adolescents were 10 (Time 1 [T1]), 12 (T2), 14 (T3), and 16 (T4) years of age, according to the World Health Organization's definition of the beginning age of adolescence.^16^ A total of 3171 pairs of adolescents and their caregivers participated in the baseline survey (T1). Of these, 3007 pairs in T2 (follow-up rate: 94.8%), 2667 pairs in T3 (84.1%), and 2616 pairs in T4 (82.5%) participated in the follow-up surveys. To examine the trajectories of psychopathological and behavioral problems, data from the 2344 pairs who participated in all four surveys were utilized.

TTC is a joint study of three institutions and has been approved by the ethics committees: Tokyo Metropolitan Institute of Medical Science (12–35), the University of Tokyo (10,057), and the Graduate University for Advanced Studies (2,012,002). Written informed consent was obtained from the primary caregivers at every time point. The Strengthening the Reporting of Observational Studies in Epidemiology (STROBE) reporting guidelines were followed.

### Measures

#### Choice of primary measures

To evaluate the trajectories of comprehensive psychopathological and behavioral problems from both adolescents' and caregivers' perspective, we collected data via widely used self-report questionnaires in the four time points (Table 1, Appendix p 3). Depressive symptoms/anxiety and psychotic-like experiences were assessed by both adolescents and caregivers. Caregivers assessed adolescents’ obsessive-compulsive symptoms, dissociative symptoms, sociality problems, hyperactivity/inattention, conduct problems, somatic symptoms, and withdrawal. To assess the above symptoms, the following scales were used: the Strength and Difficulty Questionnaire (SDQ),^17^ the Child Behavior Checklist (CBCL),^18^, ^19^, ^20^, ^21^ the Short Mood and Feelings Questionnaire (SMFQ),^22^ and the Diagnostic Interview Schedule for Children (DISC-C).^23^ Adolescents responded to single questions about desire to be slim,^24^ self-harm,^25^ and suicidal ideation.^26^ Z-scores were then calculated for each assessment by standardizing the data through T1–T4. A higher z-score indicates more severe problems. While it is possible to cluster based on item-level data, we opted for subscale-level input variables to balance interpretability, precision, computational feasibility, and minimization of bias toward specific symptom domains.

**Table 1:** Symptoms and assessments utilized for clustering.

Symptoms	Respondent	Assessments	Scores	Reference	Missing
Depression	Adolescent	SMFQ	0–26	Angold et al., 2005	–
Depression/Anxiety	Caregiver	SDQ	0–10	Goodman et al., 2006	–
Psychotic-like experiences	Adolescent	DISC-C	0–10	Costello et al., 1985	–
	Caregiver	CBCL	0–8	Lengua et al., 2001	T3
Obsession/Compulsion	Caregiver	CBCL	0–4	Ivarsson et al., 2008	T3
Dissociation	Caregiver	CBCL	0–6	Sim et al., 2005	–
Sociality problem	Caregiver	SDQ	0–10	Goodman et al., 2006	–
Hyperactivity/Inattention	Caregiver	SDQ	0–10	Goodman et al., 2006	–
Conduct problem	Caregiver	SDQ	0–10	Silva et al., 2015	–
Somatic symptom	Caregiver	CBCL	0–18	Achenbach et al., 1991	T3
Withdrawal	Caregiver	CBCL	0–18	Achenbach et al., 1991	T3
Desire to be slim	Adolescent	One query	0–3	Sugimoto et al., 2020	–
Self-harm	Adolescent	One query	0–1	Tanaka et al., 2023	T1
Suicidal ideation	Adolescent	One query	0–3	Ando et al., 2018	T1

Abbreviations: CBCL, the Child Behavior Checklist; DISC-C, Diagnostic Interview Schedule for Children; SDQ, the Strength and Difficulty Questionnaire; SMFQ, the Short Mood and Feelings Questionnaire; T1, wave at age 10; T3, wave at age 14.

#### Characteristics of participants

We aimed to comprehensively use available participant characteristic data as possible predictor candidates, referencing factors that have previously been demonstrated to be associated with transdiagnostic psychopathological trajectories.^9^, ^10^, ^11^^,^^27^ In addition, to investigate unidentified associations, we examined adolescent–caregiver relationships, an area of focus in this cohort study.^15^ The characteristic variables were obtained mainly at T1 and included aspects such as adolescent characteristics, perinatal and early childhood environment, caregiver characteristics, and family environment and relationships. Data collection was primarily conducted through self-report questionnaires separately filled out by both adolescents and caregivers, supplemented with interviews and physical measurements by investigators. Detailed definitions of the characteristic variables can be found in the Appendix (p 9). Information on pregnancy and early childhood was obtained from the Maternal and Child Health Handbook, which is widely used in Japan to document the health status of mothers and children at the time, thereby minimizing recall bias.

### Data analysis

#### Clustering with a deep learning-based model

**Fig. 1:**
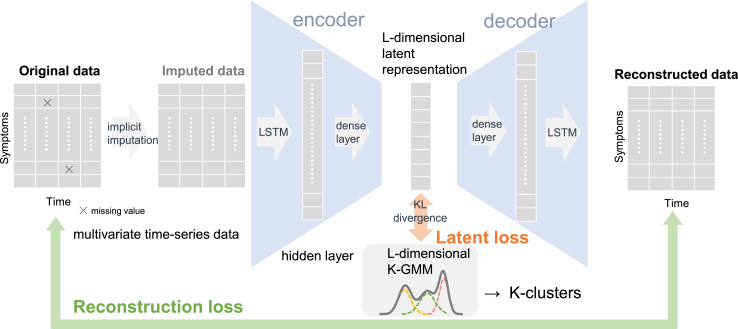
**The Architecture of Variational Deep Embedding with Recurrence (VaDER).** This figure illustrates the VaDER model's process for clustering multivariate trajectories into K groups. Beginning with implicit mean imputation for missing values, the data is then encoded into a latent L-dimensional vector. The latent representation has a mixture of K L-dimensional Gaussian distributions (Gaussian mixture model, GMM) as its prior. A decoder reconstructs the time-series data, while the model minimizes a loss function comprising reconstruction loss for effective data recovery and latent loss for regularizing the latent representation. The model is trained to minimize this loss function while learning imputed value weights and Gaussian distribution probabilities for each individual. VaDER thus integrates feature extraction, missing value imputation, and clustering into a single model.

To cluster the trajectories of multiple psychopathological and behavioral problems, a deep learning-based clustering model, variational deep embedding with recurrence (VaDER) architecture, was utilized (Fig. 1). In general, deep learning models can approximate any shape of trajectory. VaDER is an unsupervised classifier of multivariate time-series data, capable of handling missing values and a limited number of time points.^14^ It has been validated by accurately recovering clusters compared to other widely known mathematical or machine learning models. VaDER is based on variation deep embedding (VaDE). VaDE is an application of auto-encoder, a neural network widely used for dimensionality compression and feature extraction by making the input and output the same (minimization of reconstruction loss). VaDE represents the dimensionality-compressed middle layer (latent representation) as a Gaussian mixture model (GMM) (minimization of latent loss) and performs clustering based on which Gaussian distribution individuals are most likely to belong. VaDER can handle time-series data by incorporating the long short-term memory (LSTM), a neural network that can learn fluctuation patterns of multiple assessments, into VaDE. Overall, VaDER enables efficient and high-performance processing by simultaneously learning missing value imputation, feature extraction, and clustering within a single model.

The determination of the optimal hyperparameters and performing clustering with VaDER was conducted according to the following procedure (Appendix pp 4, 6–8, 24). First, we performed grid search to determine optimal hyperparameters, such as setting the latent representation dimensions to eight, based on minimizing reconstruction loss and generating a specified number of clusters. In this process, 10-fold cross-validation was repeated 20 times with the number of clusters set to 1 and the loss function set to reconstruction loss only. Hyperparameter combinations that resulted in fewer clusters than the pre-specified number were excluded. Next, the number of clusters was determined to be five by repeating 2-fold cross-validation 20 times using prediction strength as the criterion for clustering. During this process, cluster reproducibility and interpretability were considered. Finally, clustering by VaDER was performed for 100 epochs with the sum of reconstruction loss and latent loss as the loss function, under the predetermined hyperparameters and number of clusters.

To fully utilize the available information before VaDER clustering, MissForest imputation was employed specifically for scales where only a partial number of responses were missing. One hundred decision trees were generated, and predictions were made using the responses for each question item prior to calculating the overall scale score. The scale score was calculated without rounding to the nearest whole number. Imputation for a particular scale was not conducted if no responses were provided for the scale. As a result, the percentage of missing values decreased from 7.4% to 6.7%, excluding those that were not asked, for the assessments (Appendix p 5).

#### Multinomial logistic regression

To identify early predictor of psychopathological trajectory, adolescents' and familial characteristics obtained mainly at T1 were considered as predictor candidates of cluster membership (Appendix p 9). Predictor candidates were selected based on significant group differences, with the expectation that their contribution to the prediction model would be substantial. One-way analysis of variance (ANOVA) was employed for continuous variables, and the chi-square test was utilized for categorical variables. Since the tests were intended to explore and screen predictor candidates of cluster membership, multiple testing corrections were not implemented, minimizing type II error. Variables with a p-value of <0.05 in these tests were included as independent variables of multinomial logistic regression, where the outcome variable was cluster membership identified with VaDER. This multiple regression model was designed to assess the extent to which each variable uniquely predicts cluster membership by mutually adjusting for all other variables. Therefore, no adjustments for potential confounding factors were made, as such adjustments would only be necessary when the focus is specifically on the relationship between two particular variables. Continuous independent variables were standardized. The variance inflation factor (VIF) was used to assess the multicollinearity of the independent variables, and Nagelkerke's R-squared was used to assess the fit of the model. A two-sided significance level for the regression was set at 0.05.

Missing values in the predictors were addressed with multiple imputation with chained equations (MICE) under the assumption that the data were missing at random. Missing values were at most 11% for maternal smoking during pregnancy and 2.2% for predictors overall. The imputation procedure included independent variables and an outcome variable. The number of imputed datasets was set to 100 and the results were integrated according to Rubin's rule.

#### Sensitivity analysis

Several sensitivity analyses were conducted to verify the robustness of the results. These included clustering of all the participants in T1 and clustering without MissForest imputation. Additionally, the binary variable of self-harm was input as a numeric value of 0 or 1 for clustering, without standardization. Finally, regression analysis was conducted to predict cluster membership using only complete predictor candidate data.

#### Analytic software

Data were analyzed from August 2022 to February 2023. The R version 4.2.1 and Python version 3.8.13 (Python software Foundation) were utilized for clustering, statistical analysis, and missing value imputation. The VaDER algorithm, which is publicly available,^14^ was utilized.

### Role of the funding source

The funder of the study had no role in study design, data collection, data analysis, data interpretation, or writing of the report.

## Results

**Fig. 2:**
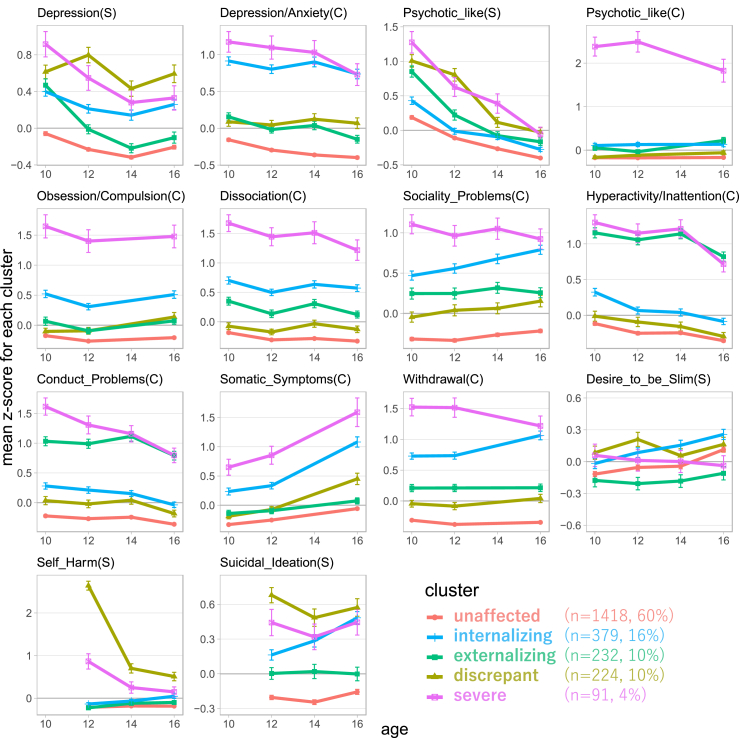
**Average trajectories of five clusters for psychopathological and behavioral problems**. This figure presents the average trajectories of the five clusters identified by deep learning-based clustering for each of the 14 psychopathological and behavioral problems. The vertical axis represents the standardized z-scores of the symptoms, with higher values indicating greater severity. It should be noted that the vertical scale is aligned to match the z-score range for each symptom. The plotted points show the mean center of standardized scores for each cluster, and error bars indicate standard errors. Assessments are denoted by (S) for self-assessment by adolescents and (C) for caregiver assessment.

Of the 2344 adolescents who participated in all four survey time points, 1095 (47%) were female (Table 2). As demonstrated in Fig. 2, the trajectories of psychopathological and behavioral problems were clustered into five groups (Appendix pp 11–12, 25). To facilitate understanding, we named the clusters according to their characteristics. Raw scores of the scales by cluster were presented in the Appendix (pp 13–14). The largest cluster, the *unaffected* group, comprised 1418 (60.5%) of the 2344 adolescents. In this cluster, the z-score of psychopathological and behavioral problems was below the overall average at almost all time points, indicating that neither the adolescents themselves nor their caregivers perceived any mental or behavioral problems. The second largest cluster was the *internalizing* group, comprising 379 (16.2%) adolescents. Adolescents in this cluster tended to exhibit persistent internalizing symptoms such as depression/anxiety and sociality problems. Especially, somatic symptoms, withdrawal, and suicidal ideations were worsening during the study period. However, in this group, psychotic-like experiences, hyperactivity/inattention, conduct problems, and self-harm were less prominent compared to other symptoms. The *discrepant* group, in which 232 pairs (9.9%) exhibited subjective symptoms such as depression and psychotic-like experiences, despite few problems identified by the caregiver's evaluation. Additionally, the z-scores of self-harm and suicidal ideation were the highest among all the clusters. The *externalizing* group included 224 (9.6%) adolescents who exhibited hyperactivity/inattention and/or conduct problems but otherwise experienced few problems. The smallest cluster was the *severe* group, to which 91 (3.9%) adolescents belonged. They continued to exhibit chronically severe problems on almost all symptom assessments by both adolescents and their caregivers. In particular, they had caregiver-reported psychotic-like experiences and symptoms of obsession/compulsion that are rarely seen in other clusters. Across all groups, there is an increase in somatic symptoms and a decrease in psychotic-like experiences.

**Table 2:** Baseline characteristics.

Characteristic	Overall (n = 2344)
Female sex	1095 (47%)
Age in months	122.1 (3.3)
Child intelligence quotient (IQ)	108 (14)
Child has any siblings	1935 (83%)
Educational background of mother	
High school or less	358/2331 (15%)
Vocational school or two-year college	1027/2331 (44%)
University	946/2331 (41%)
Educational background of father	
High school or less	381/2247 (17%)
Vocational school or two-year college	299/2247 (13%)
University	1567/2247 (70%)
Annual household income	
0–4.99 million yen	427/2262 (19%)
5–9.99 million yen	1139/2262 (50%)
≥10 million yen	696/2262 (31%)
Primary caregiver	
Mother	2322 (99%)
Father	21 (0.9%)
Other	1 (<0.1%)
Caregiver with foreign nationality	50 (2.1%)
Bereavement of caregivers	21 (0.9%)

Data are n (%) or mean (SD).

**Fig. 3:**
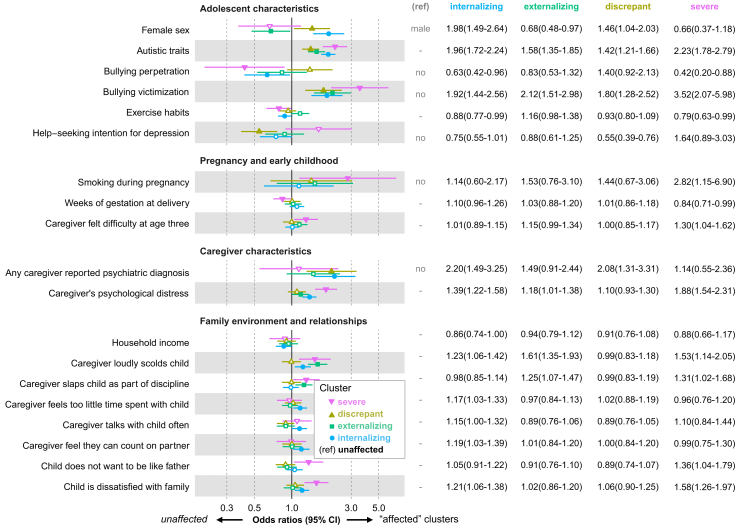
**Results of multinomial logistic regression showing cluster membership predictors and effect sizes**. This figure displays the results of a multinomial logistic regression model that examines the predictors of cluster membership and their effect sizes. The outcome variable is the membership in one of the four “affected” clusters, with the *unaffected* cluster serving as a reference. Independent variables include predictor candidates that exhibit inter-cluster differences. For categorical independent variables, the OR and its 95% CI are plotted for each variable and cluster, with the reference category shown on the right side of the figure. For continuous independent variables, the reference column shows “-”, and the OR and 95% CI are plotted for a one standard deviation increase in the variable. Filled markers represent significant OR estimates (p < 0.05), while blank markers indicate non-significant estimates. Several predictor candidates were adjusted as independent variables in the model but are not shown, as they did not significantly predict cluster membership: maternal low age, paternal low age, maternal education, paternal education, caregiver arranges for child to never fail, caregiver tells she/he loves child, caregiver praises child, caregiver's consistency in discipline, partner's consistency in discipline, discipline policy consistency between caregivers, caregiver thinks they have good relationship with child, child wants to be like mother.

The demographic characteristics of the sample population including psychiatric diagnosis, both as a whole and by cluster, are presented in the Appendix (pp 15–18). Variables that demonstrated significant inter-cluster differences through each ANOVA or chi-square test were considered predictor candidates of cluster membership. VIF was less than 1.8 for all variables, and the R-squared of this model was 0.341. Fig. 3 illustrates the results of multinomial logistic regression, in which cluster membership was the outcome variable, with the *unaffected* cluster serving as the reference (Appendix pp 19–22, 26). Females were more likely to be assigned to the *internalizing* (OR 1.98 [95% CI 1.49–2.64]) and *discrepant* (1.46 [1.04–2.03]) clusters, whereas males were more likely to be assigned to the *externalizing* cluster (1.47 [1.03–2.11]). Adolescents with stronger autistic traits (*internalizing* 1.96 [1.72–2.24]; *externalizing* 1.58 [1.35–1.85]; *discrepant* 1.42 [1.21–1.66]; *severe* 2.23 [1.78–2.79]) and those who had experienced bullying victimization (*internalizing* 1.92 [1.44–2.56]; *externalizing* 2.12 [1.51–2.98]; *discrepant* 1.80 [1.28–2.52]; *severe* 3.52 [2.07–5.98]) were more likely to be assigned to the “affected” clusters. The *discrepant* cluster membership was significantly predicted by avoiding seeking help for depression (1.83 [1.31–2.55]) and caregiver's psychiatric diagnosis (2.08 [1.31–3.31]). Lastly, the *severe* cluster membership was significantly predicted by maternal smoking during pregnancy (2.82 [1.15–6.90]), shorter weeks of gestation at delivery (1.19 [1.01–1.41]), poorer exercise habits (1.27 [1.01–1.60]), not bullying others (2.40 [1.14–5.03]), not wanting to be like father (1.36 [1.04–1.79]), adolescent's dissatisfaction with family (1.58 [1.26–1.97]), caregiver-perceived difficulty in parenting at age three (1.30 [1.04–1.62]), and caregiver's psychological distress (1.88 [1.54–2.31]). Warm parenting, characterized by frequently praising adolescents or telling them they are important, and the educational background of the caregivers were not predictive of cluster membership.

The results were robust in the sensitivity analysis: the inclusion of participants who became untraceable during the follow-up surveys, the clustering without standardizing binary variables, and the omission of missing value imputation for psychopathological assessments in clustering and for predictors in regression analysis (Appendix pp 27–30).

## Discussion

Utilizing a deep learning-based clustering model, VaDER, this study identified five distinct clusters based on the six-year longitudinal course of psychopathological and behavioral problems measured by multiple informants during adolescence. The five clusters are: *unaffected*, with minimal psychopathological symptoms; *internalizing* and *externalizing*, with strong symptoms that correspond to internalizing and externalizing symptoms, identified as major factors in previous studies; *severe*, with the persistent strong presentation of all symptoms; and *discrepant*, characterized by more severe symptoms on the adolescent's self-report assessment than on the caregiver's assessment. In addition, the results revealed that factors such as adolescent and caregiver characteristics, environmental factors during pregnancy, traumatic experiences, and familial relationships predict psychopathological trajectories.

Approximately 40% of adolescents were classified into four “affected” clusters. This aligns closely with the prevalence of mental disorders diagnosed in the general adolescent population,^3^ suggesting these groups face clinically meaningful problems. The identification of groups exhibiting primarily internalizing and externalizing factors, as well as a high psychopathology cluster, was consistent with expectations based on previous research.^28^ However, possibly due to limitations in the number of clusters, thought problems were not clearly clustered. It is understandable that only the caregivers' underestimation discrepancy group was identified, given that all the adolescent problems were not always reported to their parents.^29^ Moreover, numerous predictors were commonly found, such as stronger autistic traits and bullying victimization. Corroborating this, bullying victimization has been linked with the p factor in early adolescents,^30^ and despite historical beliefs to the contrary, a relationship between autistic traits and the p factor has also been suggested.^31^ Although these predictors may be merely manifestations of underlying causes, these predictors potentially influence the p factor, thereby impacting an adolescent's susceptibility to diverse mental health problems.

Adolescents in the *severe* group continued to exhibit particularly serious problems from early adolescence. Compared to previous studies,^9^, ^10^, ^11^ this study identified several novel predictors of *severe* trajectory including shorter weeks of gestation at delivery, caregiver-perceived difficulty in parenting at age three, stronger autistic traits, poorer exercise habits, not bullying others, and adolescent's dissatisfaction with family. In the context of bullying perpetration, OR estimates for *severe* cluster membership in simple multinomial logistic regression were greater than one (OR 1.60 [95% CI 0.91–2.82]; Appendix p 26); this result does not contradict previous findings suggesting that bullying perpetration predicts total psychopathology.^32^ Mutual adjustment in the multiple regression may have negated interaction between bullying perpetration and victimization,^33^ implying that factors unique to bullying perpetration may not necessarily exacerbate severe psychopathology. Furthermore, the *severe* group was uniquely characterized by indicators from pregnancy and early childhood emerging as significant predictors. Additionally, this group had considerably large proportion of adolescents diagnosed with neurodevelopmental disorders: 10% with autism spectrum disorder and 11% with attention deficit hyperactivity disorder. Therefore, this group could be described as “early neurodevelopmentally affected,” emphasizing the need for research and interventions which target this group throughout early childhood and adolescence.

The cluster with the highest risk for self-harm and suicidal ideation was the *discrepant* group, where adolescents' subjective psychopathological and behavioral problems were overlooked by their caregivers. Historically, discrepancies among informants have been perceived as a sign of uncertainty, leading to the underestimation of adolescents' psychopathological problems.^34^ However, our findings emphasize the importance of recognizing such discrepancies as valuable information for identifying adolescents at high risk. We advocate for future studies to utilize information from multiple informants including adolescents themselves and to pay attention to the discrepancies. Notably, both reluctances to seek help and caregiver's psychiatric diagnosis predicted membership in this group. Caregiver's mental disorders could influence adolescent psychopathology through not only genetic factors but parenting environment.^35^ It is possible that caregivers' own struggles could impair their ability to effectively care for their adolescents, or may divert attention away from the adolescents' issues, making it challenging for both caregivers and other surrounding adults to notice the adolescents' difficulties.^36^ Rather than solely relying on parents for an adolescent's care, a broader community effort is necessary. In this context, educators and community workers should proactively offer support and listen to adolescents facing difficulties, such as through regular mental health check-ins at schools and the development of community resources for free counseling.

The data-driven approach employed in this study serves as a milestone, bridging past and future research in the field of adolescent psychopathology. The replication of previously established internalizing and externalizing factors demonstrated the validity of the deep learning model in the analysis of psychopathological and behavioral problems. Additionally, this methodology introduced two key insights into psychiatry: first, it enabled classifications of psychopathological development without assumption regarding trajectory shape; second, it addressed comorbidity trajectories based on multiple informants, leading to the identification of *discrepant* cluster. Ultimately, this study contributes to advancing our understanding of the complex and diverse psychopathology of adolescents, enriching the field of psychiatry.

This study is subject to several limitations. Firstly, the inclusion of adolescents who were able to participate in all surveys might have induced selection bias and overfitting towards lower severity. However, these effects are deemed minimal given the high follow-up rate and the robust results of the sensitivity analyses. Secondly, although anxiety could contribute to the burden on adolescent mental health, the measurement of subjective anxiety was weak in this study because the SMFQ specifically focuses on core depressive symptoms. However, this does not necessitate a change in the interpretation that the distress of adolescents in the discrepant cluster was overlooked, since caregivers evaluated both depressive symptoms and anxiety with the subscale of emotional symptoms in the SDQ. Thirdly, although the process of determining the number of clusters is based on indices such as prediction strength, arbitrariness such as interpretability cannot be eliminated. For example, the *externalizing* cluster was absorbed by other clusters when the number of clusters was set to four. As the *externalizing* cluster's demographics and predictors were differentiated from those of other clusters, however, it was considered to be a meaningful group that reflected the real world. Fourthly, it is possible that symptoms such as psychotic-like experiences and self-harm, which are infrequent and tend to have a high z-score, had a large impact on the clustering. However, we also obtained robust clustering results in a sensitivity analysis where self-harm was input as binary instead of as a z-score, suggesting the effect of high z-scores is limited. Fifthly, the prediction model using multiple regression with predictors obtained in early adolescence has limitations. It does not account for the influence of time-variant predictors, thereby preventing us from examining certain questions, such as the impact of time-varying factors on the decrease in psychotic-like experiences and self-harm within the discrepant group (Appendix p 31). Additionally, the model's effectiveness depends on the variables included; for instance, if information on perinatal depression were available, it could have emerged as a stronger predictor than maternal smoking. Sixthly, our study lacks an independent sample to validate the replicability of the data-driven results. Finally, the generalizability of our findings needs to be investigated, since most of the participants were Japanese adolescents living in Tokyo, an Asian metropolis. Particularly, previous research has indicated that Japan has relatively higher levels of parent-child disagreement regarding adolescent mental health problems,^37^ warranting the need for future studies to examine the applicability of our results to other countries and regions.

In summary, utilizing a deep learning model that incorporates four-timepoint multiple assessments by both adolescents and their caregivers, we were able to cluster the six-year trajectories of psychopathological and behavioral problems in adolescence. Approximately 40% of adolescents belonged to the clusters with some psychopathological or behavioral problems, commonly predicted by stronger autistic traits and bullying victimization. The predictors of the *severe* group who continued to exhibit chronically severe problems on almost all symptoms included shorter weeks of gestation at delivery, caregiver-perceived difficulty in parenting at age three, poorer exercise habits, not bullying others, and adolescent's dissatisfaction with family. The *discrepant* cluster where adolescent subjective psychopathological problems were overlooked by caregivers exhibited the highest risks for self-harm and suicidal ideation. Proactive societal attention toward adolescents is warranted whose suicidality is overlooked and who have difficulty in seeking help.

## Contributors

DN, AU, AN, KK and SA contributed to the study concept and design. KK, AN, SY, and SA obtained research funding. AN, KK and SA supervised the conduct of the study. All authors contributed to taking data from the cohort. SU provided statistical advice. DN conducted and is responsible for the data analysis. DN drafted the manuscript, and all authors contributed substantially to its revision. SA has full access to all the data in the study and takes responsibility for the integrity of the data and the accuracy of the data analysis.

## Data sharing statement

The raw data and analytic codes supporting the conclusions of this article will be made available with publication by the corresponding author for a specified purpose after approval of proposal.

## Declaration of interests

SA received honoraria for lectures from Takeda Pharmaceuticals, Sumitomo Pharma, Shionogi & Co., Ltd., Janssen Pharmaceutical. The rest authors have no conflicts of interest to disclose.
