# Supplementary material for: Identify adolescents' help-seeking intention on suicide through self- and caregiver's assessments of psychobehavioral problems: deep clustering of the Tokyo TEEN Cohort study
Source: Lancet Reg Health West Pac. 2023 Dec 13;43:100979. doi: 10.1016/j.lanwpc.2023.100979 (PMC10920037; doi:10.1016/j.lanwpc.2023.100979)
Supplement: Supplementary Material [file mmc1.docx]

**Appendix**

**Identify adolescents’ help-seeking intention on suicide through self- and caregiver’s assessments of psychobehavioral problems: deep clustering of the Tokyo TEEN Cohort study**

[STROBE Statement—Checklist of items that should be included in reports of *cohort studies* 2](#_Toc147520370)

[eMethods 3](#_Toc147520371)

[**Measures** 3](#_Toc147520372)

[**Searching for optimal hyperparameters and the number of clusters, and executing the clustering procedure** 4](#_Toc147520373)

[eTable 1: The number of missing before and after MissForest imputation 5](#_Toc147520374)

[eTable 2: Grid search for optimal hyperparameters 6](#_Toc147520375)

[eTable 3: Prediction strength for determination of the number of clusters 8](#_Toc147520376)

[eTable 4: Definitions of characteristic variables 9](#_Toc147520377)

[eTable 5: Mean z-scores and standard errors of psychopathological problems in the five clusters 11](#_Toc147520378)

[eTable 6: Mean raw scores and standard deviations or frequency of psychopathological problems in the five clusters and overall 13](#_Toc147520379)

[eTable 7: Demographic characteristics both as a whole and by cluster 15](#_Toc147520380)

[eTable 8: Adolescent psychiatric diagnosis at age 16 by cluster 18](#_Toc147520381)

[eTable 9: Results of multinomial logistic regression 19](#_Toc147520382)

[eFigure 1: Flowchart of participant recruitment 23](#_Toc147520383)

[eFigure 2: Prediction strength for each number of clusters 24](#_Toc147520384)

[eFigure 3: Average trajectories of five cluster with unified y-scales for problems rated by both the adolescent and the caregiver 25](#_Toc147520385)

[eFigure 4: Crude results of univariable multinomial logistic regression at age 10 26](#_Toc147520386)

[eFigure 5: Result of clustering all the participants in T1 as sensitivity analysis (n = 3171) 27](#_Toc147520387)

[eFigure 6: Result of clustering without MissForest imputation as sensitivity analysis (n = 2344) 28](#_Toc147520388)

[eFigure 7: Result of clustering using 0/1 binary input for self-harm as sensitivity analysis (n = 2344) 29](#_Toc147520389)

[eFigure 8: Result of multinomial logistic regression with complete antecedent data as sensitivity analysis (n = 1530) 30](#_Toc147520390)

[eFigure 9: Crude results of univariable multinomial logistic regression at ages 12, 14, or 16 31](#_Toc147520391)

[eReferences 32](#_Toc147520392)

# STROBE Statement—Checklist of items that should be included in reports of *cohort studies*

|  | Item No | Recommendation | Page No |
| --- | --- | --- | --- |
| **Title and abstract** | 1 | (*a*) Indicate the study’s design with a commonly used term in the title or the abstract | 1–2 |
|  |  | (*b*) Provide in the abstract an informative and balanced summary of what was done and what was found | 2 |
| Introduction | | | |
| Background/rationale | 2 | Explain the scientific background and rationale for the investigation being reported | 3–6 |
| Objectives | 3 | State specific objectives, including any prespecified hypotheses | 6 |
| Methods | | | |
| Study design | 4 | Present key elements of study design early in the paper | 7 |
| Setting | 5 | Describe the setting, locations, and relevant dates, including periods of recruitment, exposure, follow-up, and data collection | 7 |
| Participants | 6 | (*a*) Give the eligibility criteria, and the sources and methods of selection of participants. Describe methods of follow-up | 7 |
|  |  | (*b*) For matched studies, give matching criteria and number of exposed and unexposed | - |
| Variables | 7 | Clearly define all outcomes, exposures, predictors, potential confounders, and effect modifiers. Give diagnostic criteria, if applicable | 7–8, S3, S9–10 |
| Data sources/ measurement | 8* | For each variable of interest, give sources of data and details of methods of assessment (measurement). Describe comparability of assessment methods if there is more than one group | 7–8, S3, S9–10 |
| Bias | 9 | Describe any efforts to address potential sources of bias | 9 |
| Study size | 10 | Explain how the study size was arrived at | 7 |
| Quantitative variables | 11 | Explain how quantitative variables were handled in the analyses. If applicable, describe which groupings were chosen and why | 8–9, S3, S9–10 |
| Statistical methods | 12 | (*a*) Describe all statistical methods, including those used to control for confounding | 8–10 |
|  |  | (*b*) Describe any methods used to examine subgroups and interactions | 9 |
|  |  | (*c*) Explain how missing data were addressed | 9 |
|  |  | (*d*) If applicable, explain how loss to follow-up was addressed | 8–10 |
|  |  | (*e*) Describe any sensitivity analyses | 9–10 |
| Results | | |  |
| Participants | 13* | (a) Report numbers of individuals at each stage of study—eg numbers potentially eligible, examined for eligibility, confirmed eligible, included in the study, completing follow-up, and analysed | 7, S23 |
|  |  | (b) Give reasons for non-participation at each stage | 7, S23 |
|  |  | (c) Consider use of a flow diagram | S23 |
| Descriptive data | 14* | (a) Give characteristics of study participants (eg demographic, clinical, social) and information on exposures and potential confounders | 11–12, 21, S11–18 |
|  |  | (b) Indicate number of participants with missing data for each variable of interest | 9, S5 |
|  |  | (c) Summarise follow-up time (eg, average and total amount) | 7 |
| Outcome data | 15* | Report numbers of outcome events or summary measures over time | 11, S11–18 |

| Main results | 16 | (*a*) Give unadjusted estimates and, if applicable, confounder-adjusted estimates and their precision (eg, 95% confidence interval). Make clear which confounders were adjusted for and why they were included | 11–12, S19–22, S26 |
| --- | --- | --- | --- |
|  |  | (*b*) Report category boundaries when continuous variables were categorized | S9–10 |
|  |  | (*c*) If relevant, consider translating estimates of relative risk into absolute risk for a meaningful time period | - |
| Other analyses | 17 | Report other analyses done—eg analyses of subgroups and interactions, and sensitivity analyses | 12, S26-31 |
| Discussion | | | |
| Key results | 18 | Summarise key results with reference to study objectives | 13 |
| Limitations | 19 | Discuss limitations of the study, taking into account sources of potential bias or imprecision. Discuss both direction and magnitude of any potential bias | 14–15 |
| Interpretation | 20 | Give a cautious overall interpretation of results considering objectives, limitations, multiplicity of analyses, results from similar studies, and other relevant evidence | 13–15 |
| Generalisability | 21 | Discuss the generalisability (external validity) of the study results | 15 |
| Other information | | | |
| Funding | 22 | Give the source of funding and the role of the funders for the present study and, if applicable, for the original study on which the present article is based | 10, 16 |

*Give information separately for exposed and unexposed groups.

# eMethods

### **Measures**

#### SDQ

Depression/anxiety, hyperactivity/inattention, conduct problem, and sociality problems were assessed utilizing the Strength and Difficulty Questionnaire (SDQ), which was completed by primary caregivers in the self-report questionnaires from T1 to T4.[^1^](https://sciwheel.com/work/citation?ids=5392629&pre=&suf=&sa=0&dbf=0) The SDQ comprises multiple subscales, such as those used to measure emotional symptoms (depression/anxiety), conduct problems, hyperactivity/inattention, and peer problems (sociality problems). Each of these subscales consists of five items which have possible three response choices: “Certainly True,” “Somewhat True,” and “Not True.” These are scored as 2, 1, and 0 points, respectively, and the scores for each item are summed to obtain a subscale score ranging from 0 to 10.

#### CBCL

Psychotic-like experiences, obsession/compulsion, dissociation, somatic symptoms, and withdrawal were assessed utilizing the Child Behavior Checklist (CBCL), for which primary caregivers provided self-report responses in the form of questionnaires.[^2^](https://sciwheel.com/work/citation?ids=14334512&pre=&suf=&sa=0&dbf=0) Responses were scored according to a three-point scale, with “Very true or Often True” receiving a score of 2, “Somewhat or Sometimes True” receiving a score of 1, and “Not True” receiving a score of 0. The CBCL subscale was utilized to score somatic symptoms and withdrawal, each with a score from 0 to 18 points. The subscales for psychotic-like experiences,[^3,4^](https://sciwheel.com/work/citation?ids=13432042,13432041&pre=&pre=&suf=&suf=&sa=0,0&dbf=0&dbf=0) obsession/compulsion,[^5^](https://sciwheel.com/work/citation?ids=13432056&pre=&suf=&sa=0&dbf=0) and dissociation[^6,7^](https://sciwheel.com/work/citation?ids=7505752,3677659&pre=&pre=&suf=&suf=&sa=0,0&dbf=0&dbf=0) were also utilized, with scores ranging from 0-8 points for psychotic-like experiences (visual hallucination, auditory hallucination, strange behavior, strange ideas), 0-4 points for obsession/compulsion (obsessions, compulsions), and 0-6 points for dissociation (confused, daydreams, stares into space). The four-item DSM-oriented Psychotic Symptoms Scale (DOPSS) approach[^3^](https://sciwheel.com/work/citation?ids=13432042&pre=&suf=&sa=0&dbf=0) has been demonstrated to have superior discriminating power in identifying psychotic symptoms in adolescents, compared to the 15-item thought problems subscale.[^4^](https://sciwheel.com/work/citation?ids=13432041&pre=&suf=&sa=0&dbf=0) This caregiver-reported assessment does not require a 'description of the experience'. Data from T1, T2, and T4, when the CBCL was included in the survey, were used for the assessment of the above symptoms, with the dissociation subscale being the only one available at all four time points as only a small portion of the CBCL was investigated at T3.

#### SMFQ

Subjective symptoms of depression and anxiety were evaluated utilizing the Short Mood and Feelings Questionnaire (SMFQ), a self-report assessment instrument for depression.[^8^](https://sciwheel.com/work/citation?ids=14334519&pre=&suf=&sa=0&dbf=0) Each of the 13 items within the questionnaire offered three response options. The scores for each item were cumulatively totaled to yield final score raging from 0-26 points, with higher scores indicating greater severity of depression.

#### DISC-C

To assess subjectively perceived psychotic-like experiences, we used five questions from the schizophrenia section of the Diagnostic Interview Schedule for Children (DISC-C),[^9,10^](https://sciwheel.com/work/citation?ids=14493676,14497662&pre=&pre=&suf=&suf=&sa=0,0) with an added question on visual hallucinations. They included mind-reading (“Have other people ever read your mind?”), receiving messages (“Have you ever had messages sent just to you through TV or radio?”), spying (“Have you ever thought that people are following or spying on you?”), auditory hallucinations (“Have you ever heard voices or sounds that no one else can hear?”), and visual hallucinations (“Have you ever seen things that other people could not see?”). Each item provided three potential responses, "Yes, definitely," "Maybe," and "No, never," which were scored as 2, 1, and 0 points, respectively. The scores for each item were subsequently summed to generate an overall score ranging from 0 to 10. Data were available at T1 through T4.

#### Single questions

The subjective desire to be slim,[^11^](https://sciwheel.com/work/citation?ids=12593272&pre=&suf=&sa=0&dbf=0) self-harm behaviors,[^12^](https://sciwheel.com/work/citation?ids=14608021&pre=&suf=&sa=0&dbf=0) and suicidal ideation[^13^](https://sciwheel.com/work/citation?ids=12593254&pre=&suf=&sa=0&dbf=0) were each evaluated based on responses to a solitary query. The desire to be slim was determined through responses to the question, "Would you like to be slimmer than you are now?" and suicidal ideation was determined through responses to the question "Do you think it is useless to live now?" Respondents were prompted to answer each of these questions using a four-point format. Each response was scored on a scale ranging from 0 (not applicable) to 3 (applicable). Self-harm behaviors were scored on a scale of 0 or 1, with 0 representing "no" and 1 representing "yes" or "more than once" in response to the question, "Have you intentionally harmed yourself within the past year?" Data were available for T1-T4 for inclination towards attaining a lean physique, T2–T4 for self-harm behaviors, and suicidal tendencies.

### **Searching for optimal hyperparameters and the number of clusters, and executing the clustering procedure**

The determination of the optimal learning conditions (hyperparameters) for performing clustering with VaDER was conducted according to the following procedure: In accordance with Jong et al.,[^14^](https://sciwheel.com/work/citation?ids=9532154&pre=&suf=&sa=0&dbf=0) a grid search was performed using the following parameter combinations to minimize reconstruction loss. Each parameter was selected within following range: learning rate = (10^-1^, 10^-2^, 10^-3^, 10^-4^); mini-batch size = (2^4^, 2^5^, 2^6^, 2^7^); number of nodes in hidden layers = (2^0^, 2^1^, 2^2^, 2^3^, 2^4^, 2^5^). As the number of clusters in the latent space, K, was set to 1 and the latent loss was not included in the loss function, only the encoder/decoder reconstruction performance was evaluated. To minimize the instability of results due to initial values and to prevent overlearning, the average of 20 iterations of 10-fold cross-validation was employed. However, if the number of dimensions in the latent representation is too high, the "curse of dimensionality" will occur, making feature extraction by dimensionality reduction impossible and rendering appropriate clustering infeasible. Therefore, the number of nodes in hidden layers was searched within the range of "14 assessments × 4 time points = 56 dimensions" or less. In the process of determining the number of clusters, K, hyperparameter combinations in which the number of clusters formed was less than the number of clusters specified in advance were excluded (As a result, the hyperparameter combinations which the number of second hidden layer was 2^4^ were excluded). Finally, the optimal hyperparameters were determined as: learning rate = 10^-3^, mini batch size = 2^5^, number of nodes in hidden layers = (2^5^, 2^3^).

Subsequently, the hyperparameters were fixed to the determined values and the search for the optimal number of clusters, K, was conducted. Prediction strength was used as a measure of the clustering model, and the difference between that in the null model with randomly labeled clusters and that in the target model was evaluated by repeating the 2-fold cross-validation 20 times. Among the number of clusters with significantly larger prediction strength than the null model, the number of clusters was determined to be 5, considering cluster reproducibility and interpretability. For interpretability, one of the conditions considered was that the size of each cluster be 50 or more.[^15^](https://sciwheel.com/work/citation?ids=11597600&pre=&suf=&sa=0&dbf=0)

Finally, clustering by VaDER was performed using the determined hyperparameters and the number of clusters. The number of epochs in the "pre-fit" phase, which is the pre-tuning of the encoder/decoder with reconstruction loss as the only loss function, and the number of epochs in the "fit" phase, in which learning and clustering are performed by adding the latent loss to the loss function, were set to 100 for each. An 8-dimensional latent representation was obtained from the time series data of 14 assessments x 4 time points, and the data was assigned to 5 clusters at the end.

# eTable 1: The number of missing before and after MissForest imputation

|  | Before |  |  |  | After |  |  |  |
| --- | --- | --- | --- | --- | --- | --- | --- | --- |
|  | T1 | T2 | T3 | T4 | T1 | T2 | T3 | T4 |
| Depression/Anxiety(S) | 152 | 338 | 467 | 492 | 8 | 297 | 436 | 459 |
| Depression/Anxiety(C) | 7 | 11 | 15 | 16 | 0 | 1 | 4 | 2 |
| Psychotic_like(S) | 88 | 330 | 450 | 475 | 7 | 287 | 437 | 468 |
| Psychotic_like(C) | 6 | 134 | - | 190 | 1 | 125 | - | 173 |
| Obsession/Compulsion(C) | 7 | 130 | - | 187 | 1 | 125 | - | 173 |
| Dissociation(C) | 6 | 132 | 167 | 187 | 1 | 125 | 161 | 173 |
| Sociality_Problems(C) | 9 | 12 | 10 | 20 | 0 | 1 | 4 | 2 |
| Hyperactivity/Inattention(C) | 10 | 14 | 11 | 13 | 0 | 1 | 4 | 2 |
| Conduct_Problems(C) | 8 | 12 | 16 | 10 | 0 | 1 | 4 | 2 |
| Somatic_Symptoms(C) | 52 | 163 | - | 215 | 1 | 125 | - | 173 |
| Withdrawal(C) | 9 | 136 | - | 196 | 1 | 125 | - | 173 |
| Desire_to_be_Slim(S) | 65 | 339 | 443 | 466 | 65 | 339 | 443 | 466 |
| Self_Harm(S) | - | 338 | 449 | 474 | - | 338 | 449 | 474 |
| Suicidal_Ideation(S) | - | 313 | 457 | 470 | - | 313 | 457 | 470 |

# eTable 2: Grid search for optimal hyperparameters

| **learning_rate** | **batch_size** | **n_hidden1** | **n_hidden2** | **train_reconstruction_loss** | **1.96*SE** | **test_reconstruction_loss** | **1.96*SE** |
| --- | --- | --- | --- | --- | --- | --- | --- |
| 0.001 | 16 | 32 | 16 | 16.87 | 0.07 | 19.93 | 0.05 |
| 0.001 | 32 | 32 | 16 | 17.80 | 0.07 | 20.29 | 0.04 |
| 0.01 | 128 | 32 | 16 | 15.63 | 0.07 | 20.81 | 0.07 |
| 0.001 | 64 | 32 | 16 | 19.07 | 0.08 | 21.08 | 0.05 |
| 0.01 | 64 | 32 | 16 | 15.25 | 0.05 | 21.28 | 0.06 |
| 0.01 | 16 | 32 | 16 | 15.21 | 0.05 | 21.43 | 0.05 |
| 0.01 | 32 | 32 | 16 | 15.15 | 0.07 | 21.47 | 0.05 |
| 0.001 | 128 | 32 | 16 | 20.74 | 0.08 | 22.27 | 0.06 |
| 1.00E-04 | 16 | 32 | 16 | 25.67 | 0.16 | 26.54 | 0.14 |
| 1.00E-04 | 32 | 32 | 16 | 28.82 | 0.21 | 29.55 | 0.14 |
| **0.001** | **32** | **32** | **8** | **25.93** | **0.13** | **29.57** | **0.06** |
| 0.001 | 64 | 32 | 8 | 27.00 | 0.11 | 29.69 | 0.07 |
| 0.1 | 128 | 32 | 16 | 27.18 | 0.84 | 29.78 | 0.74 |
| 0.001 | 16 | 32 | 8 | 25.03 | 0.16 | 29.80 | 0.06 |
| 0.01 | 128 | 16 | 8 | 27.05 | 0.11 | 29.92 | 0.06 |
| 0.001 | 16 | 16 | 8 | 28.23 | 0.23 | 29.92 | 0.06 |
| 0.01 | 64 | 16 | 8 | 26.58 | 0.11 | 30.04 | 0.07 |
| 0.01 | 32 | 16 | 8 | 26.37 | 0.10 | 30.11 | 0.06 |
| 0.001 | 128 | 32 | 8 | 28.32 | 0.10 | 30.12 | 0.06 |
| 0.01 | 16 | 16 | 8 | 26.46 | 0.12 | 30.18 | 0.05 |
| 0.001 | 32 | 16 | 8 | 28.86 | 0.12 | 30.30 | 0.06 |
| 0.001 | 64 | 16 | 8 | 29.65 | 0.12 | 30.84 | 0.06 |
| 0.001 | 128 | 16 | 8 | 30.92 | 0.10 | 31.77 | 0.06 |
| 0.1 | 128 | 16 | 8 | 29.25 | 0.12 | 31.91 | 0.08 |
| 1.00E-04 | 16 | 32 | 8 | 31.15 | 0.19 | 32.04 | 0.08 |
| 0.01 | 128 | 32 | 8 | 22.95 | 0.07 | 32.04 | 0.07 |
| 0.01 | 64 | 32 | 8 | 22.53 | 0.07 | 32.65 | 0.06 |
| 0.01 | 16 | 32 | 8 | 22.78 | 0.12 | 32.79 | 0.10 |
| 0.01 | 32 | 32 | 8 | 22.47 | 0.11 | 32.96 | 0.09 |
| 0.1 | 128 | 32 | 8 | 28.45 | 0.11 | 33.13 | 0.08 |
| 1.00E-04 | 32 | 32 | 8 | 32.63 | 0.19 | 33.34 | 0.13 |
| 1.00E-04 | 64 | 32 | 16 | 32.95 | 0.19 | 33.56 | 0.13 |
| 0.1 | 64 | 16 | 8 | 31.78 | 0.18 | 33.77 | 0.10 |
| 1.00E-04 | 16 | 16 | 8 | 35.07 | 0.37 | 35.60 | 0.23 |
| 1.00E-04 | 64 | 32 | 8 | 35.62 | 0.19 | 36.22 | 0.16 |
| 0.1 | 64 | 32 | 8 | 34.44 | 0.81 | 36.75 | 0.62 |
| 1.00E-04 | 32 | 16 | 8 | 38.10 | 0.18 | 38.60 | 0.19 |
| 1.00E-04 | 128 | 32 | 16 | 39.87 | 0.28 | 40.10 | 0.25 |
| 1.00E-04 | 128 | 32 | 8 | 41.64 | 0.33 | 41.82 | 0.33 |
| 1.00E-04 | 64 | 16 | 8 | 42.84 | 0.41 | 43.27 | 0.36 |
| 0.1 | 32 | 16 | 8 | 42.40 | 0.56 | 43.35 | 0.54 |
| 1.00E-04 | 128 | 16 | 8 | 50.09 | 0.42 | 50.11 | 0.44 |

Abbreviations: n_hidden1, the number of nodes in the first hidden layer; n_hidden2, the number of nodes in the second hidden layer; train_reconstruction_loss, reconstruction loss in the test data; test_reconstruction_loss, reconstruction loss in the test data.

# eTable 3: Prediction strength for determination of the number of clusters

| **k** | **learning_rate** | **batch_size** | **n_hidden1** | **n_hidden2** | **prediction_strength** | **1.96*SE** | **prediction_strength_null** | **1.96*SE** |
| --- | --- | --- | --- | --- | --- | --- | --- | --- |
| 2 | 0.001 | 32 | 32 | 8 | 1.1E-01 | 6.3E-03 | 1.0E-01 | 2.9E-03 |
| 3 | 0.001 | 32 | 32 | 8 | 1.9E-02 | 4.0E-03 | 1.6E-02 | 2.7E-03 |
| 4 | 0.001 | 32 | 32 | 8 | 1.4E-02 | 3.1E-03 | 9.9E-03 | 1.4E-03 |
| **5** | **0.001** | **32** | **32** | **8** | **1.0E-02** | **1.5E-03** | **6.3E-03** | **1.1E-03** |
| 6 | 0.001 | 32 | 32 | 8 | 7.2E-03 | 1.2E-03 | 3.9E-03 | 8.1E-04 |
| 7 | 0.001 | 32 | 32 | 8 | 5.8E-03 | 8.6E-04 | 2.9E-03 | 5.4E-04 |
| 8 | 0.001 | 32 | 32 | 8 | 4.7E-03 | 8.2E-04 | 2.1E-03 | 3.3E-04 |
| 9 | 0.001 | 32 | 32 | 8 | 3.0E-03 | 6.3E-04 | 1.3E-03 | 1.9E-04 |
| 10 | 0.001 | 32 | 32 | 8 | 2.4E-03 | 4.4E-04 | 9.9E-04 | 1.8E-04 |
| 11 | 0.001 | 32 | 32 | 8 | 1.9E-03 | 3.3E-04 | 6.9E-04 | 1.3E-04 |
| 12 | 0.001 | 32 | 32 | 8 | 1.9E-03 | 3.0E-04 | 5.9E-04 | 1.2E-04 |
| 13 | 0.001 | 32 | 32 | 8 | 1.3E-03 | 2.3E-04 | 4.3E-04 | 9.5E-05 |
| 14 | 0.001 | 32 | 32 | 8 | 1.2E-03 | 3.2E-04 | 3.2E-04 | 8.8E-05 |
| 15 | 0.001 | 32 | 32 | 8 | 9.9E-04 | 2.0E-04 | 2.3E-04 | 5.0E-05 |

Abbreviations: k, the number of clusters; n_hidden1, the number of nodes in hidden layer 1; n_hidden2, the number of nodes in the second hidden layer; prediction_strength_null, the value of prediction strength in a null model.

# eTable 4: Definitions of characteristic variables

| **Adolescent characteristics** |  |
| --- | --- |
| Female sex | Primary caregivers responded by choosing male or female at T1. |
| Autistic traits | AQ-J-10,[^16^](https://sciwheel.com/work/citation?ids=14402415&pre=&suf=&sa=0&dbf=0) shortened version of the 50-item Autism-spectrum Quotient: AQ,[^17^](https://sciwheel.com/work/citation?ids=876072&pre=&suf=&sa=0&dbf=0) validated in Japanese. Scored from 0-10 as a continuous variable.  Primary caregiver’s report at T2 was utilized, since AQ is not measured at T1 and trait is assumed to be time-invariant. |
| Bullying perpetration | Adolescent-report at T1.  Dummy variable where 1 is the child who reported experiencing any of the following in the past 2 months. “I have ostracized, ignored, or spoken ill of others behind their backs,” “I have made fun of others, or uttered bad words, threats, or other nasty remarks,” “I have lightly bumped, hit, or kicked others under the pretense of play,” “I have hit, slapped, or kicked others strongly,” “I have taken or broken someone's money or belongings.” |
| Bullying victimization | Adolescent-report at T1.  Dummy variable where 1 is the child who reported experiencing any of the following in the past 2 months.  “I was ostracized, ignored, or spoken ill of behind my back,” “I was made fun of, or had bad words, threats, or other nasty remarks directed at me,” “I was lightly bumped, hit, or kicked under the pretense of play,” “I was hit, slapped, or kicked strongly,” “I have had money or belongings taken or broken.” |
| Exercise habits | Adolescent-report at T1. Continuous variable.  Question: “Do you play sports or engage in physical activities outside of school?”  The multiple-choice and scores were as follows;“4. almost everyday; 3. more than once a week; 2. more than once a month; 1. less than once a month; 0. Never.” |
| Help-seeking intention for depression | Adolescent-report at T1.  Dummy variable with 1 being the child who answered "no one" to the question “If you were in the same situation as Taro, would you talk to someone about it?” (A sentence is presented in advance that indicates that A child named Taro is depressed.) |
| Child IQ | Intelligence quotient (IQ) of adolescents was estimated from two subsets (Information and Picture Completion) of the Wechsler Intelligence Scale for Children (WISC-III)[^18^](https://sciwheel.com/work/citation?ids=12157963&pre=&suf=&sa=0&dbf=0), which was assessed by investigators at T1. |
| BMI | Body mass index = Body weight (kg) / height (m)2  Body weight and height of adolescents were measured by the investigators at T1. |
| Lefty | Dummy variable with 1 if the adolescents were responded that their dominant hand was left. |
| **Pregnancy and early childhood** |  |
| Smoking during pregnancy | Dummy variable with 1 if the Maternal and Child Health Handbook states that the mother smoked during pregnancy. The Maternal and Child Health Handbook is a handbook widely used in Japan to record the health status of mothers and children during pregnancy and after delivery. |
| Weeks of gestation at delivery | The number of weeks of gestation at the time of delivery as indicated in the Maternal and Child Health Handbook. |
| Caregiver felts difficulty at age three | To the question "Do you find it difficult to raise your child?" which was answered by the caregiver when the child was 3 years old (Maternal and Child Health Handbook), we scored those who answered "yes" as 2, "indescribable" as 1, and "no" as 0. |
| Pregnancy complications | Dummy variable with 1 if the Maternal and Child Health Handbook states that there were any complications during pregnancy. |
| Alcohol during pregnancy | Dummy variable with 1 if the Maternal and Child Health Handbook states that the mother took alcohol during pregnancy. |
| Birth weight | The birth weight of adolescents as indicated in the Maternal and Child Health Handbook. |
| **Caregiver characteristics** |  |
| Paternal low age | Paternal age was below 35 at T1. The age was reported by primary caregivers. |
| Maternal low age | Maternal age was below 35 at T1. The age was reported by primary caregivers. |
| Paternal education | Primary caregiver’s report at T1. Continuous variables as follows: 1 for junior high school graduation or less, 2 for high school dropout, 3 for high school graduation, 4 for vocational school/junior college graduation, 5 for 4-year college graduation, and 6 for graduate school/6-year college graduation. |
| Maternal education | Same as above. |
| Caregiver psychiatric diagnosis | Dummy variable with 1 if the primary caregiver indicated that the primary caregiver or his/her partner has been diagnosed with one of the following mental illnesses; depression, anxiety disorder, bipolar disorder, or schizophrenia. |
| Caregiver psychological distress | Caregiver-report at T1. The Kessler Psychological Distress Scale (K6) is scored from 0 to 6.[^19^](https://sciwheel.com/work/citation?ids=3609491&pre=&suf=&sa=0&dbf=0) |
| Mother IQ | IQ of mothers was estimated with Japanese Adult Reading Test (JART)[^20^](https://sciwheel.com/work/citation?ids=14402419&pre=&suf=&sa=0&dbf=0) assessed at T1. |
| Primary caregiver | The respondent as a primary caregiver at T1. |
| Caregiver with foreign nationality | Dummy variable with 1 if the nationality of either caregiver was other than Japan at T1. |
| **Family environment and relationships** |  |
| Household income | Caregiver-reported annual household income at T1 as a continuous variable. |
| Caregiver scolds child loudly | Caregiver-reported at T1.  Question: “Do you scold your child loudly?”  Continuous variables as follow: 1for rarely, 2 for sometimes, 3 for often, 4 for always. |
| Caregiver slaps child as part of discipline | Caregiver-reported at T1.  Question: “Do you slap your child as part of discipline?”  Continuous variables as follow: 1for rarely, 2 for sometimes, 3 for often, 4 for always. |
| Caregiver tells she/he loves child | Caregiver-reported at T1.  Question: “Do you tell your children that you love them or care about them?”  Continuous variables as follow: 1for rarely, 2 for sometimes, 3 for often, 4 for always. |
| Caregiver praises child | Caregiver-reported at T1.  Question: “Do you praise your child?”  Continuous variables as follow: 1for rarely, 2 for sometimes, 3 for often, 4 for always. |
| Caregiver arranges for child to never fail | Caregiver-reported at T1.  Question: “Do you try and make arrangements so that your child never fails?”  Continuous variables as follow: 1for rarely, 2 for sometimes, 3 for often, 4 for always. |
| Caregiver consistency in discipline | Caregiver-reported at T1.  Question: “Do you consistently praise and scold your child?”  Continuous variables as follow: 1for almost never consistent, 2 for not very consistent, 3 for mostly consistent, 4 for always consistent. |
| Partner consistency in discipline | Caregiver-reported at T1.  Question: “Does your partner consistently praise and scold your child?”  Continuous variables as follow: 1for almost never consistent, 2 for not very consistent, 3 for mostly consistent, 4 for always consistent. |
| Discipline policy consistency between caregivers | Caregiver-reported at T1.  Question: “Is your discipline policy consistent with your partner's?”  Continuous variables as follow: 1for almost never consistent, 2 for not very consistent, 3 for mostly consistent, 4 for always consistent. |
| Caregiver feels too little time spent with child | Caregiver-reported at T1.  Question: “How do you feel about time spent with your child?”  Continuous variables as follow: 1 for too long, 2 for longer than long enough, 3 for just long enough, 4 for nowhere near long enough, 5 for not long enough at all. |
| Caregiver talks with child often | Caregiver-reported at T1.  Question: “Do you talk with your child often?”  Continuous variables as follow: 1 for sometimes do not talk for more than 1 day, 2 for always talk at least once a day, 3 for talk for about 1 hour a day, 4 for talk for more than 2 hours a day. |
| Caregiver thinks they have good relationship with child | Caregiver-reported at T1.  Question: “Do you have good relationship with your child?”  Continuous variables as follow: 1for not very good, 2 for not so good, 3 for fair to good, 4 for very good. |
| Caregiver feels she/he can count on partner | Caregiver-reported at T1.  Question: “Is your partner helpful in times of need with regard to child rearing?”  Continuous variables as follow: 1 for partner is rarely helpful, 2 for partner is not very helpful, 3 for partner is usually helpful, 4 for partner is always helpful. |
| Child does not want to be like father | Adolescent-reported at T1.  Question: “Do you want to be like your father in the future?”  Continuous variables as follow: 1for agree, 2 for somewhat agree, 3 for somewhat disagree, 4 for disagree. |
| Child does not want to be like mother | Adolescent-reported at T1.  Question: “Do you want to be like your mother in the future?”  Continuous variables as follow: 1for agree, 2 for somewhat agree, 3 for somewhat disagree, 4 for disagree. |
| Child is dissatisfied with family | Adolescent-reported at T1.  Question: “How do you feel about your family? Circle "1" if you are very satisfied and circle "7" if you are not satisfied at all.”  Responses on a Likert scale were used as continuous variables. |
| Child has any Siblings | Dummy variable with 1 if the number of children reported by caregivers was more than one at T1. |
| Bereavement of caregivers | Dummy variable with 1 if the adolescent has experienced the bereavement of either caregiver at T1. |
| Caregiver tells child to solve problems by him/herself | Caregiver-reported at T1.  Question: “Do you tell your children to try to solve problems by themselves instead of relying on others right away, even when things are hard or difficult?”  Continuous variables as follow: 1 for true, 2 for somewhat true, 3 for not very true, 4 for not true. |
| Caregiver tries to protect child from any difficulties | Caregiver-reported at T1.  Question: “Do you try to protect your child from all the difficulties that arise in life?”  Continuous variables as follow: 1for rarely, 2 for sometimes, 3 for often, 4 for always. |
| Child feels father helps with homework | Adolescent-reported at T1.  Question: “Does your father help you with your homework?”  Dummy variable with 1 if the adolescent responded that the father helped with homework. |
| Child feels mother helps with homework | Adolescent-reported at T1.  Question: “Does your mother help you with your homework?”  Dummy variable with 1 if the adolescent responded that the mother helped with homework. |

# eTable 5: Mean z-scores and standard errors of psychopathological problems in the five clusters

Depression(S)

|  | mean10 | se10 | mean12 | se12 | mean14 | se14 | mean16 | se16 |
| --- | --- | --- | --- | --- | --- | --- | --- | --- |
| unaffected | -0.058 | 0.021 | -0.230 | 0.017 | -0.316 | 0.017 | -0.206 | 0.021 |
| discrepant | 0.614 | 0.074 | 0.797 | 0.084 | 0.432 | 0.084 | 0.593 | 0.096 |
| externalizing | 0.469 | 0.068 | -0.012 | 0.049 | -0.218 | 0.049 | -0.102 | 0.060 |
| internalizing | 0.398 | 0.050 | 0.212 | 0.047 | 0.143 | 0.055 | 0.259 | 0.057 |
| severe | 0.916 | 0.138 | 0.548 | 0.134 | 0.280 | 0.137 | 0.330 | 0.132 |

Depression/Anxiety(C)

|  | mean10 | se10 | mean12 | se12 | mean14 | se14 | mean16 | se16 |
| --- | --- | --- | --- | --- | --- | --- | --- | --- |
| unaffected | -0.156 | 0.020 | -0.295 | 0.019 | -0.362 | 0.018 | -0.398 | 0.018 |
| discrepant | 0.088 | 0.061 | 0.045 | 0.062 | 0.123 | 0.076 | 0.069 | 0.073 |
| externalizing | 0.155 | 0.055 | -0.017 | 0.047 | 0.038 | 0.057 | -0.149 | 0.053 |
| internalizing | 0.913 | 0.056 | 0.802 | 0.058 | 0.901 | 0.067 | 0.738 | 0.065 |
| severe | 1.173 | 0.141 | 1.096 | 0.157 | 1.030 | 0.161 | 0.729 | 0.148 |

Psychotic_like(S)

|  | mean10 | se10 | mean12 | se12 | mean14 | se14 | mean16 | se16 |
| --- | --- | --- | --- | --- | --- | --- | --- | --- |
| unaffected | 0.187 | 0.026 | -0.111 | 0.021 | -0.267 | 0.016 | -0.401 | 0.012 |
| discrepant | 1.006 | 0.090 | 0.805 | 0.091 | 0.115 | 0.072 | -0.027 | 0.066 |
| externalizing | 0.852 | 0.081 | 0.222 | 0.073 | -0.076 | 0.055 | -0.165 | 0.054 |
| internalizing | 0.429 | 0.054 | -0.013 | 0.042 | -0.093 | 0.039 | -0.277 | 0.030 |
| severe | 1.275 | 0.157 | 0.627 | 0.137 | 0.388 | 0.140 | -0.058 | 0.105 |

Psychotic_like(C)

|  | mean10 | se10 | mean12 | se12 | mean14 | se14 | mean16 | se16 |
| --- | --- | --- | --- | --- | --- | --- | --- | --- |
| unaffected | -0.173 | 0.018 | -0.176 | 0.016 | NA | NA | -0.167 | 0.017 |
| discrepant | -0.164 | 0.035 | -0.113 | 0.046 | NA | NA | -0.060 | 0.045 |
| externalizing | 0.057 | 0.059 | -0.034 | 0.046 | NA | NA | 0.222 | 0.072 |
| internalizing | 0.107 | 0.050 | 0.131 | 0.047 | NA | NA | 0.135 | 0.055 |
| severe | 2.380 | 0.217 | 2.490 | 0.236 | NA | NA | 1.827 | 0.262 |

Obsession/Compulsion(C)

|  | mean10 | se10 | mean12 | se12 | mean14 | se14 | mean16 | se16 |
| --- | --- | --- | --- | --- | --- | --- | --- | --- |
| unaffected | -0.174 | 0.022 | -0.265 | 0.016 | NA | NA | -0.207 | 0.018 |
| discrepant | -0.104 | 0.054 | -0.093 | 0.057 | NA | NA | 0.136 | 0.075 |
| externalizing | 0.066 | 0.069 | -0.094 | 0.050 | NA | NA | 0.073 | 0.057 |
| internalizing | 0.521 | 0.061 | 0.308 | 0.053 | NA | NA | 0.510 | 0.063 |
| severe | 1.646 | 0.193 | 1.400 | 0.189 | NA | NA | 1.478 | 0.189 |

Dissociation(C)

|  | mean10 | se10 | mean12 | se12 | mean14 | se14 | mean16 | se16 |
| --- | --- | --- | --- | --- | --- | --- | --- | --- |
| unaffected | -0.185 | 0.021 | -0.307 | 0.017 | -0.284 | 0.018 | -0.328 | 0.017 |
| discrepant | -0.075 | 0.057 | -0.173 | 0.048 | -0.033 | 0.061 | -0.128 | 0.056 |
| externalizing | 0.350 | 0.066 | 0.136 | 0.064 | 0.308 | 0.071 | 0.123 | 0.060 |
| internalizing | 0.701 | 0.060 | 0.499 | 0.054 | 0.636 | 0.062 | 0.572 | 0.057 |
| severe | 1.675 | 0.143 | 1.445 | 0.153 | 1.512 | 0.187 | 1.217 | 0.173 |

Sociality_Problems(C)

|  | mean10 | se10 | mean12 | se12 | mean14 | se14 | mean16 | se16 |
| --- | --- | --- | --- | --- | --- | --- | --- | --- |
| unaffected | -0.318 | 0.021 | -0.334 | 0.021 | -0.265 | 0.021 | -0.217 | 0.021 |
| discrepant | -0.046 | 0.063 | 0.039 | 0.069 | 0.063 | 0.068 | 0.153 | 0.071 |
| externalizing | 0.247 | 0.068 | 0.248 | 0.068 | 0.318 | 0.068 | 0.257 | 0.062 |
| internalizing | 0.470 | 0.056 | 0.558 | 0.058 | 0.677 | 0.059 | 0.790 | 0.056 |
| severe | 1.107 | 0.118 | 0.964 | 0.128 | 1.052 | 0.132 | 0.924 | 0.125 |

Hyperactivity/Inattention(C)

|  | mean10 | se10 | mean12 | se12 | mean14 | se14 | mean16 | se16 |
| --- | --- | --- | --- | --- | --- | --- | --- | --- |
| unaffected | -0.116 | 0.024 | -0.251 | 0.023 | -0.245 | 0.021 | -0.356 | 0.019 |
| discrepant | -0.012 | 0.070 | -0.093 | 0.066 | -0.157 | 0.062 | -0.295 | 0.054 |
| externalizing | 1.154 | 0.070 | 1.056 | 0.069 | 1.139 | 0.068 | 0.821 | 0.065 |
| internalizing | 0.324 | 0.053 | 0.067 | 0.048 | 0.040 | 0.050 | -0.086 | 0.044 |
| severe | 1.299 | 0.108 | 1.149 | 0.130 | 1.210 | 0.127 | 0.717 | 0.110 |

Conduct_Problems(C)

|  | mean10 | se10 | mean12 | se12 | mean14 | se14 | mean16 | se16 |
| --- | --- | --- | --- | --- | --- | --- | --- | --- |
| unaffected | -0.223 | 0.021 | -0.269 | 0.020 | -0.244 | 0.021 | -0.363 | 0.018 |
| discrepant | 0.034 | 0.069 | -0.021 | 0.065 | 0.041 | 0.066 | -0.183 | 0.056 |
| externalizing | 1.033 | 0.076 | 0.991 | 0.076 | 1.116 | 0.082 | 0.791 | 0.073 |
| internalizing | 0.281 | 0.052 | 0.212 | 0.054 | 0.154 | 0.049 | -0.036 | 0.045 |
| severe | 1.616 | 0.144 | 1.307 | 0.150 | 1.159 | 0.137 | 0.795 | 0.122 |

Somatic_Symptoms(C)

|  | mean10 | se10 | mean12 | se12 | mean14 | se14 | mean16 | se16 |
| --- | --- | --- | --- | --- | --- | --- | --- | --- |
| unaffected | -0.331 | 0.013 | -0.254 | 0.015 | NA | NA | -0.058 | 0.020 |
| discrepant | -0.188 | 0.044 | -0.073 | 0.052 | NA | NA | 0.450 | 0.098 |
| externalizing | -0.141 | 0.054 | -0.094 | 0.051 | NA | NA | 0.074 | 0.052 |
| internalizing | 0.234 | 0.059 | 0.334 | 0.056 | NA | NA | 1.082 | 0.088 |
| severe | 0.650 | 0.136 | 0.854 | 0.153 | NA | NA | 1.589 | 0.244 |

Withdrawal(C)

|  | mean10 | se10 | mean12 | se12 | mean14 | se14 | mean16 | se16 |
| --- | --- | --- | --- | --- | --- | --- | --- | --- |
| unaffected | -0.312 | 0.017 | -0.381 | 0.015 | NA | NA | -0.347 | 0.017 |
| discrepant | -0.044 | 0.054 | -0.083 | 0.058 | NA | NA | 0.041 | 0.067 |
| externalizing | 0.210 | 0.058 | 0.212 | 0.059 | NA | NA | 0.215 | 0.060 |
| internalizing | 0.728 | 0.050 | 0.736 | 0.057 | NA | NA | 1.064 | 0.070 |
| severe | 1.524 | 0.143 | 1.515 | 0.156 | NA | NA | 1.219 | 0.160 |

Desire_to_be_Slim(S)

|  | mean10 | se10 | mean12 | se12 | mean14 | se14 | mean16 | se16 |
| --- | --- | --- | --- | --- | --- | --- | --- | --- |
| unaffected | -0.117 | 0.024 | -0.055 | 0.023 | -0.042 | 0.024 | 0.114 | 0.025 |
| discrepant | 0.084 | 0.062 | 0.210 | 0.067 | 0.057 | 0.065 | 0.163 | 0.067 |
| externalizing | -0.177 | 0.060 | -0.207 | 0.059 | -0.183 | 0.060 | -0.110 | 0.063 |
| internalizing | -0.017 | 0.049 | 0.085 | 0.046 | 0.154 | 0.048 | 0.257 | 0.048 |
| severe | 0.059 | 0.106 | 0.012 | 0.102 | 0.001 | 0.104 | -0.037 | 0.093 |

Self_Harm(S)

|  | mean10 | se10 | mean12 | se12 | mean14 | se14 | mean16 | se16 |
| --- | --- | --- | --- | --- | --- | --- | --- | --- |
| unaffected | NA | NA | -0.212 | 0.009 | -0.181 | 0.011 | -0.184 | 0.011 |
| discrepant | NA | NA | 2.640 | 0.104 | 0.701 | 0.108 | 0.510 | 0.100 |
| externalizing | NA | NA | -0.224 | 0.017 | -0.118 | 0.042 | -0.098 | 0.045 |
| internalizing | NA | NA | -0.134 | 0.032 | -0.065 | 0.040 | 0.048 | 0.050 |
| severe | NA | NA | 0.864 | 0.179 | 0.251 | 0.133 | 0.150 | 0.117 |

Suicidal_Ideation(S)

|  | mean10 | se10 | mean12 | se12 | mean14 | se14 | mean16 | se16 |
| --- | --- | --- | --- | --- | --- | --- | --- | --- |
| unaffected | NA | NA | -0.204 | 0.019 | -0.245 | 0.021 | -0.156 | 0.022 |
| discrepant | NA | NA | 0.680 | 0.066 | 0.485 | 0.075 | 0.573 | 0.076 |
| externalizing | NA | NA | 0.003 | 0.050 | 0.020 | 0.061 | -0.001 | 0.059 |
| internalizing | NA | NA | 0.163 | 0.045 | 0.285 | 0.054 | 0.484 | 0.054 |
| severe | NA | NA | 0.443 | 0.114 | 0.319 | 0.111 | 0.445 | 0.110 |

Abbreviations: (S), self-reported assessment by adolescents; (C), caregiver-reported assessment; mean10, mean z-score at age 10; se10, standard error of z-score at age 10; NA, not applicable.

# eTable 6: Mean raw scores and standard deviations or frequency of psychopathological problems in the five clusters and overall

|  |  | **Age 10** | **Age 12** | **Age 14** | **Age 16** |
| --- | --- | --- | --- | --- | --- |
| **Depression(S)** SMFQ  Scores 0–26 | **Overall** | 4.7 (4.5) | 3.9 (4.5) | 3.1 (4.6) | 3.8 (5.2) |
|  | **unaffected** | 3.6 (3.8) | 2.6 (3.3) | 2.0 (3.3) | 2.7 (4.1) |
|  | **discrepant** | 6.8 (5.2) | 7.8 (6.0) | 6.2 (6.2) | 7.2 (7.3) |
|  | **externalizing** | 6.1 (5.0) | 3.8 (3.8) | 2.6 (3.9) | 3.3 (4.8) |
|  | **internalizing** | 5.8 (4.6) | 5.1 (4.6) | 4.7 (5.7) | 5.4 (5.8) |
|  | **severe** | 8.3 (6.3) | 6.8 (6.4) | 5.5 (6.7) | 5.9 (6.7) |
| **Depression/Anxiety(C)**  SDQ Emotional Symptoms  Scores 0–10 | **Overall** | 1.6 (1.7) | 1.3 (1.7) | 1.3 (1.8) | 1.2 (1.7) |
|  | **unaffected** | 1.1 (1.3) | 0.8 (1.2) | 0.7 (1.2) | 0.7 (1.1) |
|  | **discrepant** | 1.5 (1.6) | 1.4 (1.6) | 1.6 (1.9) | 1.5 (1.9) |
|  | **externalizing** | 1.6 (1.4) | 1.3 (1.2) | 1.4 (1.5) | 1.1 (1.4) |
|  | **internalizing** | 2.9 (1.9) | 2.7 (1.9) | 2.9 (2.2) | 2.6 (2.2) |
|  | **severe** | 3.3 (2.3) | 3.2 (2.6) | 3.1 (2.6) | 2.6 (2.4) |
| **Psychotic-like experience(S)**  DISC-C  Scores 0–10 | **Overall** | 1.8 (1.9) | 1.2 (1.7) | 0.8 (1.4) | 0.4 (1.1) |
|  | **unaffected** | 1.4 (1.6) | 0.9 (1.4) | 0.5 (1.1) | 0.2 (0.8) |
|  | **discrepant** | 2.7 (2.2) | 2.4 (2.2) | 1.3 (1.9) | 1.0 (1.8) |
|  | **externalizing** | 2.5 (2.0) | 1.5 (2.0) | 0.9 (1.5) | 0.7 (1.5) |
|  | **internalizing** | 1.8 (1.7) | 1.0 (1.4) | 0.9 (1.4) | 0.5 (1.1) |
|  | **severe** | 3.2 (2.5) | 2.2 (2.2) | 1.8 (2.4) | 0.9 (1.9) |
| **Psychotic-like experience(C)**  CBCL Psychosis-like experience  Scores 0–8 | **Overall** | 4.2 (0.6) | 4.2 (0.6) | NA | 4.2 (0.6) |
|  | **unaffected** | 4.1 (0.4) | 4.0 (0.3) | NA | 4.0 (0.4) |
|  | **discrepant** | 4.1 (0.3) | 4.1 (0.4) | NA | 4.1 (0.4) |
|  | **externalizing** | 4.2 (0.5) | 4.1 (0.4) | NA | 4.3 (0.6) |
|  | **internalizing** | 4.2 (0.6) | 4.2 (0.5) | NA | 4.2 (0.6) |
|  | **severe** | 5.5 (1.2) | 5.6 (1.2) | NA | 5.3 (1.4) |
| **Obsession/Compulsion(C)**  CBCL Obsession Compulsion Scores 0–4 | **Overall** | 2.3 (0.6) | 2.2 (0.5) | NA | 2.3 (0.6) |
|  | **unaffected** | 2.1 (0.4) | 2.1 (0.3) | NA | 2.1 (0.4) |
|  | **discrepant** | 2.2 (0.4) | 2.2 (0.5) | NA | 2.3 (0.6) |
|  | **externalizing** | 2.3 (0.6) | 2.2 (0.4) | NA | 2.3 (0.5) |
|  | **internalizing** | 2.5 (0.6) | 2.4 (0.6) | NA | 2.5 (0.7) |
|  | **severe** | 3.1 (1.0) | 3.0 (1.0) | NA | 3.1 (1.0) |
| **Dissociation(C)**  CBCL Dissociation  Scores 0–6 | **Overall** | 3.7 (0.9) | 3.5 (0.8) | 3.6 (1.0) | 3.5 (0.9) |
|  | **unaffected** | 3.4 (0.7) | 3.3 (0.6) | 3.3 (0.7) | 3.3 (0.6) |
|  | **discrepant** | 3.5 (0.8) | 3.4 (0.7) | 3.6 (0.8) | 3.5 (0.8) |
|  | **externalizing** | 3.9 (0.9) | 3.7 (0.9) | 3.9 (1.0) | 3.7 (0.8) |
|  | **internalizing** | 4.2 (1.1) | 4.1 (1.0) | 4.2 (1.1) | 4.1 (1.0) |
|  | **severe** | 5.1 (1.2) | 5.0 (1.3) | 5.0 (1.6) | 4.8 (1.5) |
| **Sociality problem(C)**  SDQ Peer Problems  Scores 0–10 | **Overall** | 1.5 (1.6) | 1.5 (1.6) | 1.6 (1.6) | 1.7 (1.6) |
|  | **unaffected** | 1.1 (1.3) | 1.1 (1.3) | 1.2 (1.3) | 1.2 (1.3) |
|  | **discrepant** | 1.5 (1.5) | 1.6 (1.7) | 1.7 (1.6) | 1.8 (1.7) |
|  | **externalizing** | 2.0 (1.7) | 2.0 (1.7) | 2.1 (1.7) | 2.0 (1.5) |
|  | **internalizing** | 2.4 (1.8) | 2.5 (1.8) | 2.7 (1.9) | 2.9 (1.8) |
|  | **severe** | 3.4 (1.8) | 3.1 (2.0) | 3.3 (2.0) | 3.1 (1.9) |
| **Hyperactivity/Inattention(C)**  SDQ Hyperactivity Inattention Scores 0–10 | **Overall** | 3.0 (2.1) | 2.7 (2.0) | 2.7 (2.0) | 2.4 (1.8) |
|  | **unaffected** | 2.5 (1.8) | 2.2 (1.7) | 2.2 (1.6) | 2.0 (1.4) |
|  | **discrepant** | 2.7 (2.1) | 2.5 (2.0) | 2.4 (1.9) | 2.1 (1.6) |
|  | **externalizing** | 5.0 (2.1) | 4.8 (2.1) | 5.0 (2.1) | 4.3 (2.0) |
|  | **internalizing** | 3.3 (2.1) | 2.8 (1.9) | 2.8 (1.9) | 2.5 (1.7) |
|  | **severe** | 5.3 (2.1) | 5.0 (2.5) | 5.1 (2.4) | 4.1 (2.1) |
| **Conduct problem(C)**  SDQ Conduct Problems  Scores 0–10 | **Overall** | 1.8 (1.6) | 1.7 (1.5) | 1.7 (1.5) | 1.5 (1.3) |
|  | **unaffected** | 1.4 (1.2) | 1.3 (1.1) | 1.3 (1.1) | 1.1 (1.0) |
|  | **discrepant** | 1.7 (1.5) | 1.7 (1.4) | 1.8 (1.5) | 1.4 (1.2) |
|  | **externalizing** | 3.2 (1.7) | 3.2 (1.7) | 3.4 (1.9) | 2.9 (1.6) |
|  | **internalizing** | 2.1 (1.5) | 2.0 (1.5) | 1.9 (1.4) | 1.6 (1.3) |
|  | **severe** | 4.1 (2.0) | 3.6 (2.1) | 3.4 (1.9) | 2.9 (1.7) |
| **Somatic Symptom(C)**  CBCL Somatic Complaints  Scores 0–18 | **Overall** | 9.6 (1.2) | 9.7 (1.3) | NA | 10.2 (2.0) |
|  | **unaffected** | 9.3 (0.7) | 9.4 (0.9) | NA | 9.7 (1.2) |
|  | **discrepant** | 9.6 (1.0) | 9.7 (1.2) | NA | 10.6 (2.3) |
|  | **externalizing** | 9.6 (1.3) | 9.7 (1.2) | NA | 10.0 (1.2) |
|  | **internalizing** | 10.2 (1.8) | 10.4 (1.7) | NA | 11.6 (2.7) |
|  | **severe** | 10.8 (2.0) | 11.2 (2.3) | NA | 12.5 (3.6) |
| **Withdrawal(C)**  CBCL Withdrawn  Scores 0–18 | **Overall** | 10.5 (1.7) | 10.4 (1.8) | NA | 10.6 (2.1) |
|  | **unaffected** | 9.9 (1.1) | 9.7 (1.0) | NA | 9.8 (1.2) |
|  | **discrepant** | 10.4 (1.5) | 10.3 (1.6) | NA | 10.6 (1.9) |
|  | **externalizing** | 10.9 (1.6) | 10.9 (1.7) | NA | 10.9 (1.8) |
|  | **internalizing** | 11.8 (1.8) | 11.9 (2.1) | NA | 12.6 (2.6) |
|  | **severe** | 13.3 (2.5) | 13.4 (2.8) | NA | 13.0 (2.9) |
| **Desire to be slim(S)**  Original  Scores 0–3 | **Overall** | 1.3 (1.1) | 1.4 (1.2) | 1.4 (1.3) | 1.6 (1.3) |
|  | **unaffected** | 1.2 (1.1) | 1.3 (1.1) | 1.3 (1.2) | 1.6 (1.3) |
|  | **discrepant** | 1.5 (1.2) | 1.7 (1.2) | 1.5 (1.2) | 1.6 (1.3) |
|  | **externalizing** | 1.2 (1.1) | 1.1 (1.2) | 1.1 (1.2) | 1.2 (1.3) |
|  | **internalizing** | 1.4 (1.2) | 1.5 (1.2) | 1.6 (1.2) | 1.8 (1.3) |
|  | **severe** | 1.5 (1.3) | 1.4 (1.3) | 1.4 (1.3) | 1.3 (1.2) |
| **Self-harm(S)**  Original  Binary | **Overall** | NA | 225/2006 (11.2%) | 111/1895 (5.9%) | 107/1870 (5.7%) |
|  | **unaffected** | NA | 12/1198 (1.0%) | 18/1127 (1.6%) | 17/1125 (1.5%) |
|  | **discrepant** | NA | 174/216 (80.6%) | 57/198 (28.8%) | 45/190 (23.7%) |
|  | **externalizing** | NA | 1/193 (0.5%) | 7/186 (3.8%) | 8/183 (4.4%) |
|  | **internalizing** | NA | 11/320 (3.4%) | 17/307 (5.5%) | 28/302 (9.3%) |
|  | **severe** | NA | 27/79 (34%) | 12/77 (16%) | 9/70 (12.9%) |
| **Suicidal ideation(S)**  Original  Scores 0–3 | **Overall** | NA | 0.8 (0.9) | 0.8 (1.1) | 0.9 (1.1) |
|  | **unaffected** | NA | 0.6 (0.8) | 0.5 (0.9) | 0.6 (0.9) |
|  | **discrepant** | NA | 1.5 (1.0) | 1.4 (1.2) | 1.5 (1.2) |
|  | **externalizing** | NA | 0.8 (0.8) | 0.8 (1.1) | 0.8 (1.0) |
|  | **internalizing** | NA | 1.0 (0.9) | 1.2 (1.2) | 1.4 (1.1) |
|  | **severe** | NA | 1.3 (1.2) | 1.2 (1.2) | 1.4 (1.2) |

Data are mean (SD) or n/N (%). NA, not applicable; SMFQ, Short Mood and Feeling Questionnaire; SDQ, Strength and Difficulties Questionnaire; CBCL, Child Behavior Check List.

# eTable 7: Demographic characteristics both as a whole and by cluster

| **Characteristic** | **Overall, N = 2,344^1^** | **unaffected, N = 1,418^1^** | **discrepant, N = 224^1^** | **externalizing, N = 232^1^** | **internalizing, N = 379^1^** | **severe, N = 91^1^** | **p-value^2^** |
| --- | --- | --- | --- | --- | --- | --- | --- |
| **Female Sex** | 1,095 (47%) | 668 (47%) | 111 (50%) | 68 (29%) | 216 (57%) | 32 (35%) | **<0.0001** |
| **Child IQ** | 108 (14) | 108 (14) | 109 (14) | 107 (14) | 108 (15) | 108 (14) | 0.77 |
| missing | 3 | 0 | 0 | 2 | 0 | 1 |  |
| **Autistic traits** | 2.50 (1.81) | 2.09 (1.52) | 2.64 (1.92) | 3.06 (1.92) | 3.28 (2.06) | 3.97 (2.06) | **<0.0001** |
| missing | 134 | 87 | 2 | 16 | 24 | 5 |  |
| **BMI** | 16.76 (2.20) | 16.75 (2.14) | 16.71 (2.22) | 16.66 (2.11) | 16.80 (2.27) | 17.22 (2.87) | 0.31 |
| missing | 5 | 2 | 1 | 0 | 2 | 0 |  |
| **Bullying victimization** | 714 (32%) | 335 (25%) | 90 (42%) | 99 (44%) | 140 (38%) | 50 (56%) | **<0.0001** |
| missing | 83 | 52 | 10 | 7 | 13 | 1 |  |
| **Bullying perpetration** | 303 (14%) | 165 (12%) | 43 (21%) | 37 (18%) | 42 (12%) | 16 (19%) | **0.0018** |
| missing | 129 | 67 | 16 | 23 | 17 | 6 |  |
| **Help-seeking intention for depression** | 522 (23%) | 269 (19%) | 70 (32%) | 60 (27%) | 103 (28%) | 20 (23%) | **<0.0001** |
| missing | 31 | 12 | 3 | 6 | 7 | 3 |  |
| **Exercise habits** | 1.88 (0.87) | 1.82 (0.85) | 1.92 (0.88) | 1.72 (0.74) | 2.09 (0.92) | 2.16 (1.01) | **<0.0001** |
| missing | 9 | 5 | 2 | 2 | 0 | 0 |  |
| **Lefty** | 192 (8.2%) | 110 (7.8%) | 26 (12%) | 22 (9.5%) | 24 (6.3%) | 10 (11%) | 0.13 |
| missing | 4 | 3 | 0 | 0 | 0 | 1 |  |
| **Pregnancy complications** | 148 (6.3%) | 86 (6.1%) | 13 (5.8%) | 18 (7.8%) | 25 (6.6%) | 6 (6.6%) | 0.89 |
| **Smoking during pregnancy** | 99 (4.8%) | 48 (3.8%) | 10 (5.1%) | 14 (6.7%) | 16 (4.8%) | 11 (13%) | **0.0016** |
| missing | 260 | 152 | 29 | 24 | 48 | 7 |  |
| **Alcohol during pregnancy** | 554 (27%) | 318 (25%) | 57 (30%) | 59 (29%) | 98 (30%) | 22 (26%) | 0.33 |
| missing | 290 | 166 | 35 | 29 | 53 | 7 |  |
| **Weeks of gestation at delivery** | 38.93 (1.78) | 38.94 (1.81) | 38.96 (1.66) | 38.93 (1.70) | 39.04 (1.51) | 38.35 (2.61) | **0.026** |
| missing | 33 | 18 | 8 | 2 | 5 | 0 |  |
| **Birth weight** | 3,022 (422) | 3,030 (417) | 3,012 (442) | 2,995 (387) | 3,034 (407) | 2,940 (563) | 0.26 |
| missing | 26 | 16 | 4 | 3 | 2 | 1 |  |
| **Caregiver felt difficulty at age three** | 196 (8.7%) | 99 (7.3%) | 17 (7.9%) | 32 (14%) | 27 (7.5%) | 21 (24%) | **<0.0001** |
| missing | 90 | 53 | 8 | 9 | 18 | 2 |  |
| **Maternal low age** | 121 (5.2%) | 61 (4.3%) | 12 (5.4%) | 11 (4.8%) | 27 (7.2%) | 10 (11%) | **0.022** |
| missing | 9 | 6 | 0 | 1 | 2 | 0 |  |
| **Paternal low age** | 50 (2.3%) | 24 (1.8%) | 3 (1.4%) | 5 (2.3%) | 13 (3.7%) | 5 (5.7%) | **0.036** |
| missing | 127 | 65 | 17 | 11 | 30 | 4 |  |
| **Paternal education** | 4.63 (0.97) | 4.66 (0.95) | 4.66 (1.04) | 4.46 (1.12) | 4.63 (0.92) | 4.50 (0.95) | **0.033** |
| missing | 97 | 52 | 12 | 7 | 23 | 3 |  |
| **Maternal education** | 4.28 (0.80) | 4.31 (0.77) | 4.34 (0.83) | 4.19 (0.76) | 4.24 (0.85) | 4.05 (0.83) | **0.0071** |
| missing | 13 | 9 | 1 | 1 | 2 | 0 |  |
| **Mother IQ** | 107 (9) | 107 (8) | 107 (9) | 106 (9) | 107 (9) | 106 (8) | 0.74 |
| missing | 42 | 25 | 2 | 4 | 11 | 0 |  |
| **Caregiver psychological distress** | 2.92 (3.30) | 2.35 (2.93) | 2.87 (3.23) | 3.56 (3.41) | 3.89 (3.40) | 6.36 (4.68) | **<0.0001** |
| missing | 10 | 7 | 1 | 0 | 2 | 0 |  |
| **Caregiver psychiatric diagnosis** | 230 (9.8%) | 94 (6.6%) | 30 (13%) | 28 (12%) | 62 (16%) | 16 (18%) | **<0.0001** |
| **Household income** | 8.16 (2.60) | 8.32 (2.58) | 8.17 (2.57) | 7.88 (2.58) | 7.93 (2.66) | 7.43 (2.72) | **0.0014** |
| missing | 82 | 55 | 7 | 4 | 14 | 2 |  |
| **Child has any Siblings** | 1,935 (83%) | 1,185 (84%) | 181 (81%) | 189 (81%) | 307 (81%) | 73 (80%) | 0.62 |
| **Primary caregiver** |  |  |  |  |  |  | 0.89 |
| Mother | 2,322 (99%) | 1,406 (99%) | 222 (99%) | 228 (98%) | 375 (99%) | 91 (100%) |  |
| Father | 21 (0.9%) | 11 (0.8%) | 2 (0.9%) | 4 (1.7%) | 4 (1.1%) | 0 (0%) |  |
| Other | 1 (<0.1%) | 1 (<0.1%) | 0 (0%) | 0 (0%) | 0 (0%) | 0 (0%) |  |
| **Caregiver with foreign nationality** | 50 (2.1%) | 29 (2.0%) | 10 (4.5%) | 4 (1.7%) | 6 (1.6%) | 1 (1.1%) | 0.13 |
| **Bereavement of caregivers** | 21 (0.9%) | 11 (0.8%) | 2 (0.9%) | 1 (0.4%) | 7 (1.8%) | 0 (0%) | 0.24 |
| **Caregiver scolds child loudly** | 2.35 (0.82) | 2.23 (0.78) | 2.26 (0.80) | 2.78 (0.78) | 2.45 (0.84) | 2.82 (0.81) | **<0.0001** |
| missing | 9 | 5 | 1 | 1 | 2 | 0 |  |
| **Caregiver slaps child as part of discipline** | 1.47 (0.63) | 1.39 (0.58) | 1.42 (0.64) | 1.78 (0.72) | 1.49 (0.64) | 1.88 (0.74) | **<0.0001** |
| missing | 11 | 7 | 1 | 1 | 2 | 0 |  |
| **Caregiver tells she/he loves child** | 3.01 (0.93) | 3.05 (0.93) | 3.12 (0.93) | 2.77 (0.88) | 2.97 (0.95) | 2.90 (0.91) | **<0.0001** |
| missing | 4 | 1 | 0 | 1 | 1 | 1 |  |
| **Caregiver praises child** | 3.06 (0.78) | 3.12 (0.79) | 3.10 (0.76) | 2.84 (0.71) | 2.99 (0.78) | 2.86 (0.82) | **<0.0001** |
| missing | 3 | 1 | 0 | 1 | 1 | 0 |  |
| **Discipline policy consistency between caregivers** | 3.31 (0.73) | 3.37 (0.68) | 3.36 (0.80) | 3.15 (0.73) | 3.22 (0.80) | 3.05 (0.77) | **<0.0001** |
| missing | 7 | 4 | 1 | 1 | 1 | 0 |  |
| **Caregiver tells child to solve problems by him/herself** | 2.11 (0.75) | 2.09 (0.75) | 2.11 (0.76) | 2.13 (0.74) | 2.17 (0.73) | 2.10 (0.73) | 0.47 |
| missing | 3 | 1 | 0 | 1 | 1 | 0 |  |
| **Caregiver talks with child often** | 3.00 (0.79) | 3.04 (0.79) | 2.93 (0.79) | 2.84 (0.79) | 3.03 (0.80) | 2.93 (0.81) | **0.0038** |
| missing | 9 | 5 | 1 | 1 | 2 | 0 |  |
| **Caregiver thinks they have good relationship with child** | 3.47 (0.70) | 3.51 (0.72) | 3.44 (0.73) | 3.33 (0.57) | 3.46 (0.67) | 3.24 (0.66) | **0.00010** |
| missing | 11 | 6 | 1 | 2 | 2 | 0 |  |
| **Caregiver tries to protect child from any difficulties** | 2.92 (0.91) | 2.91 (0.93) | 2.96 (0.88) | 2.90 (0.91) | 2.96 (0.85) | 2.86 (0.84) | 0.73 |
| missing | 16 | 11 | 1 | 1 | 3 | 0 |  |
| **Caregiver arranges for child to never fail** | 2.23 (0.80) | 2.20 (0.81) | 2.22 (0.78) | 2.27 (0.82) | 2.31 (0.76) | 2.43 (0.75) | **0.014** |
| missing | 14 | 7 | 1 | 1 | 3 | 2 |  |
| **Caregiver consistency in discipline** | 3.00 (0.43) | 3.03 (0.41) | 3.00 (0.44) | 2.97 (0.47) | 2.94 (0.46) | 2.91 (0.46) | **0.0013** |
| missing | 5 | 3 | 0 | 1 | 1 | 0 |  |
| **Caregiver feels too little time spent with child** | 3.24 (0.70) | 3.21 (0.66) | 3.27 (0.70) | 3.26 (0.76) | 3.32 (0.74) | 3.27 (0.86) | 0.074 |
| missing | 11 | 6 | 2 | 1 | 2 | 0 |  |
| **Partner consistency in discipline** | 3.11 (0.60) | 3.17 (0.58) | 3.12 (0.61) | 3.00 (0.57) | 3.02 (0.62) | 2.90 (0.68) | **<0.0001** |
| missing | 112 | 62 | 12 | 9 | 25 | 4 |  |
| **Caregiver feels she/he can count on partner** | 3.25 (0.69) | 3.29 (0.66) | 3.25 (0.74) | 3.13 (0.71) | 3.23 (0.71) | 2.94 (0.81) | **<0.0001** |
| missing | 115 | 63 | 16 | 8 | 24 | 4 |  |
| **Child feels father helps with homework** | 1,243 (54%) | 753 (54%) | 124 (57%) | 118 (52%) | 204 (54%) | 44 (50%) | 0.79 |
| missing | 45 | 29 | 5 | 4 | 4 | 3 |  |
| **Child feels mother helps with homework** | 1,243 (71%) | 753 (71%) | 124 (73%) | 118 (67%) | 204 (72%) | 44 (69%) | 0.74 |
| missing | 595 | 359 | 55 | 57 | 97 | 27 |  |
| **Child does not want to be like father** | 2.08 (0.94) | 2.03 (0.92) | 2.00 (0.93) | 2.09 (0.93) | 2.22 (0.97) | 2.52 (0.97) | **<0.0001** |
| missing | 54 | 35 | 6 | 4 | 5 | 4 |  |
| **Child does not want to be like mother** | 1.90 (0.91) | 1.85 (0.89) | 1.85 (0.86) | 2.12 (0.98) | 1.93 (0.92) | 2.09 (0.97) | **0.0015** |
| missing | 48 | 33 | 5 | 3 | 4 | 3 |  |
| **Child dissatisfaction with family** | 1.73 (1.11) | 1.60 (1.01) | 1.76 (1.16) | 1.82 (1.15) | 1.95 (1.22) | 2.51 (1.36) | **<0.0001** |
| missing | 42 | 28 | 5 | 6 | 2 | 1 |  |
| ^1^n (%); Mean (SD) | | | | | | | |
| ^2^Pearson's Chi-squared test; One-way ANOVA | | | | | | | |

# eTable 8: Adolescent psychiatric diagnosis at age 16 by cluster

|  | **Overall**,  N = 2,344 | **unaffected**,  N = 1,418 | **discrepant**,  N = 224 | **externalizing**,  N = 232 | **internalizing**,  N = 379 | **severe**,  N = 91 | **p-value**  Fisher’s exact test |
| --- | --- | --- | --- | --- | --- | --- | --- |
| **Depression** | 24/2291  (1.0%) | 6/1384  (0.4%) | 8/220  (3.6%) | 0/228  (0%) | 10/369  (2.7%) | 0/90  (0%) | **< 0.0001** |
| **Anxiety disorder** | 33/2291  (1.4%) | 7/1385  (0.5%) | 4/219  (1.8%) | 2/228  (0.9%) | 16/369  (4.3%) | 4/90  (4.4%) | **< 0.0001** |
| **Bipolar disorder** | 6/2290  (0.3%) | 1/1385  (<0.1%) | 2/219  (0.9%) | 0/228  (0%) | 1/368  (0.3%) | 2/90  (2.2%) | **0.0062** |
| **Schizophrenia** | 5/2289  (0.2%) | 3/1384  (0.2%) | 1/219  (0.5%) | 0/227  (0%) | 1/369  (0.3%) | 0/90  (0%) | 0.74 |
| **Autism spectrum disorder** | 37/2287  (1.6%) | 10/1380  (0.7%) | 3/219  (1.4%) | 7/228  (3.1%) | 8/370  (2.2%) | 9/90  (10%) | **< 0.0001** |
| **Attention deficit hyperactivity disorder** | 51/2291  (2.2%) | 9/1383  (0.7%) | 3/219  (1.4%) | 15/228  (6.6%) | 14/370  (3.8%) | 10/91  (11%) | **< 0.0001** |
| **Learning disability** | 30/2289  (1.3%) | 7/1383  (0.5%) | 5/219  (2.3%) | 5/228  (2.2%) | 7/369  (1.9%) | 6/90  (6.7%) | **< 0.0001** |

Adolescents’ diagnosis of mental disorders was reported by primary caregivers at T4 (age 16).

# eTable 9: Results of multinomial logistic regression

| cluster | explanatory variables | estimate | std.error | OR | 95%CI_low | 95%CI_high | p.value |
| --- | --- | --- | --- | --- | --- | --- | --- |
| internalizing | (Intercept) | -1.83 | 0.17 | 0.16 | 0.12 | 0.22 | <0.0001 |
| internalizing | female sex | 0.68 | 0.15 | 1.98 | 1.49 | 2.64 | <0.0001 |
| internalizing | autistic traits | 0.67 | 0.07 | 1.96 | 1.72 | 2.24 | <0.0001 |
| internalizing | bullying perpetration | -0.46 | 0.21 | 0.63 | 0.42 | 0.96 | 0.033 |
| internalizing | bullying victimization | 0.65 | 0.15 | 1.92 | 1.44 | 2.56 | <0.0001 |
| internalizing | exercise habits | -0.13 | 0.06 | 0.88 | 0.77 | 0.99 | 0.034 |
| internalizing | help-seeking intention | -0.29 | 0.15 | 0.75 | 0.55 | 1.01 | 0.056 |
| internalizing | smoking during pregnancy | 0.13 | 0.33 | 1.14 | 0.60 | 2.17 | 0.69 |
| internalizing | weeks of gestation at delivery | 0.09 | 0.07 | 1.10 | 0.96 | 1.26 | 0.17 |
| internalizing | caregiver felt difficulty at age three | 0.01 | 0.07 | 1.01 | 0.89 | 1.15 | 0.88 |
| internalizing | caregiver psychiatric diagnosis | 0.79 | 0.20 | 2.20 | 1.49 | 3.25 | <0.0001 |
| internalizing | caregiver psychological distress | 0.33 | 0.07 | 1.39 | 1.22 | 1.58 | <0.0001 |
| internalizing | household_income | -0.15 | 0.07 | 0.86 | 0.74 | 1.00 | 0.045 |
| internalizing | caregiver scolds child loudly | 0.20 | 0.08 | 1.23 | 1.06 | 1.42 | 0.0070 |
| internalizing | caregiver slaps child as part of discipline | -0.02 | 0.07 | 0.98 | 0.85 | 1.14 | 0.82 |
| internalizing | Caregiver feels too little time spent with child | 0.15 | 0.07 | 1.17 | 1.03 | 1.33 | 0.018 |
| internalizing | Caregiver talks with child often | 0.14 | 0.07 | 1.15 | 1.00 | 1.32 | 0.046 |
| internalizing | Caregiver feels she/he can count on partner | 0.18 | 0.08 | 1.19 | 1.03 | 1.39 | 0.023 |
| internalizing | Child does not want to be like father | 0.05 | 0.08 | 1.05 | 0.91 | 1.22 | 0.46 |
| internalizing | Child dissatisfaction with family | 0.19 | 0.07 | 1.21 | 1.06 | 1.38 | 0.0048 |
| internalizing | paternal education | 0.10 | 0.08 | 1.10 | 0.95 | 1.28 | 0.21 |
| internalizing | maternal education | -0.02 | 0.07 | 0.99 | 0.86 | 1.13 | 0.83 |
| internalizing | maternal low age | 0.38 | 0.31 | 1.46 | 0.79 | 2.70 | 0.22 |
| internalizing | paternal low age | 0.14 | 0.46 | 1.15 | 0.47 | 2.84 | 0.76 |
| internalizing | Caregiver tells they love child | 0.06 | 0.08 | 1.06 | 0.90 | 1.25 | 0.50 |
| internalizing | Caregiver praises child | -0.05 | 0.08 | 0.95 | 0.81 | 1.12 | 0.54 |
| internalizing | Discipline policy consistency between caregivers | -0.07 | 0.07 | 0.93 | 0.81 | 1.08 | 0.33 |
| internalizing | Caregiver thinks they have good relationship with child | 0.03 | 0.07 | 1.03 | 0.90 | 1.18 | 0.69 |
| internalizing | Caregiver arranges for child to never fail | 0.07 | 0.07 | 1.08 | 0.95 | 1.22 | 0.27 |
| internalizing | Caregiver consistency in discipline | -0.07 | 0.07 | 0.93 | 0.82 | 1.06 | 0.29 |
| internalizing | Partner consistency in discipline | -0.15 | 0.08 | 0.86 | 0.74 | 1.00 | 0.053 |
| internalizing | Child does not want to be like mother | 0.00 | 0.08 | 1.00 | 0.86 | 1.17 | 0.97 |
| externalizing | (Intercept) | -2.07 | 0.19 | 0.13 | 0.09 | 0.18 | <0.0001 |
| externalizing | female sex | -0.39 | 0.18 | 0.68 | 0.48 | 0.97 | 0.034 |
| externalizing | autistic traits | 0.46 | 0.08 | 1.58 | 1.35 | 1.85 | <0.0001 |
| externalizing | bullying perpetration | -0.18 | 0.23 | 0.83 | 0.53 | 1.32 | 0.44 |
| externalizing | bullying victimization | 0.75 | 0.17 | 2.12 | 1.51 | 2.98 | <0.0001 |
| externalizing | exercise habits | 0.15 | 0.09 | 1.16 | 0.98 | 1.38 | 0.080 |
| externalizing | help-seeking intention | -0.13 | 0.18 | 0.88 | 0.61 | 1.25 | 0.47 |
| externalizing | smoking during pregnancy | 0.43 | 0.36 | 1.53 | 0.76 | 3.10 | 0.24 |
| externalizing | weeks of gestation at delivery | 0.03 | 0.08 | 1.03 | 0.88 | 1.20 | 0.75 |
| externalizing | caregiver felt difficulty at age three | 0.14 | 0.08 | 1.15 | 0.99 | 1.34 | 0.064 |
| externalizing | caregiver psychiatric diagnosis | 0.40 | 0.25 | 1.49 | 0.91 | 2.44 | 0.11 |
| externalizing | caregiver psychological distress | 0.16 | 0.08 | 1.18 | 1.01 | 1.38 | 0.039 |
| externalizing | household_income | -0.06 | 0.09 | 0.94 | 0.79 | 1.12 | 0.49 |
| externalizing | caregiver scolds child loudly | 0.48 | 0.09 | 1.61 | 1.35 | 1.93 | <0.0001 |
| externalizing | caregiver slaps child as part of discipline | 0.22 | 0.08 | 1.25 | 1.07 | 1.47 | 0.0052 |
| externalizing | Caregiver feels too little time spent with child | -0.03 | 0.08 | 0.97 | 0.84 | 1.13 | 0.69 |
| externalizing | Caregiver talks with child often | -0.11 | 0.08 | 0.89 | 0.76 | 1.06 | 0.18 |
| externalizing | Caregiver feels she/he can count on partner | 0.01 | 0.09 | 1.01 | 0.84 | 1.20 | 0.95 |
| externalizing | Child does not want to be like father | -0.09 | 0.09 | 0.91 | 0.76 | 1.10 | 0.33 |
| externalizing | Child dissatisfaction with family | 0.02 | 0.08 | 1.02 | 0.86 | 1.20 | 0.85 |
| externalizing | paternal education | -0.10 | 0.09 | 0.91 | 0.77 | 1.08 | 0.27 |
| externalizing | maternal education | -0.05 | 0.09 | 0.95 | 0.81 | 1.13 | 0.58 |
| externalizing | maternal low age | -0.12 | 0.40 | 0.89 | 0.40 | 1.95 | 0.77 |
| externalizing | paternal low age | -0.30 | 0.58 | 0.74 | 0.24 | 2.31 | 0.61 |
| externalizing | Caregiver tells they love child | -0.16 | 0.10 | 0.86 | 0.70 | 1.04 | 0.12 |
| externalizing | Caregiver praises child | -0.08 | 0.10 | 0.92 | 0.76 | 1.12 | 0.42 |
| externalizing | Discipline policy consistency between caregivers | -0.10 | 0.09 | 0.90 | 0.76 | 1.07 | 0.24 |
| externalizing | Caregiver thinks they have good relationship with child | -0.03 | 0.08 | 0.97 | 0.82 | 1.14 | 0.70 |
| externalizing | Caregiver arranges for child to never fail | 0.07 | 0.08 | 1.07 | 0.92 | 1.25 | 0.39 |
| externalizing | Caregiver consistency in discipline | 0.14 | 0.08 | 1.15 | 0.98 | 1.34 | 0.086 |
| externalizing | Partner consistency in discipline | -0.14 | 0.09 | 0.87 | 0.73 | 1.04 | 0.13 |
| externalizing | Child does not want to be like mother | 0.15 | 0.09 | 1.16 | 0.98 | 1.38 | 0.086 |
| discrepant | (Intercept) | -1.90 | 0.18 | 0.15 | 0.10 | 0.21 | <0.0001 |
| discrepant | female sex | 0.38 | 0.17 | 1.46 | 1.04 | 2.03 | 0.027 |
| discrepant | autistic traits | 0.35 | 0.08 | 1.42 | 1.21 | 1.66 | <0.0001 |
| discrepant | bullying perpetration | 0.33 | 0.21 | 1.40 | 0.92 | 2.13 | 0.12 |
| discrepant | bullying victimization | 0.59 | 0.17 | 1.80 | 1.28 | 2.52 | 0.00069 |
| discrepant | exercise habits | -0.07 | 0.08 | 0.93 | 0.80 | 1.09 | 0.38 |
| discrepant | help-seeking intention | -0.60 | 0.17 | 0.55 | 0.39 | 0.76 | 0.00039 |
| discrepant | smoking during pregnancy | 0.36 | 0.39 | 1.44 | 0.67 | 3.06 | 0.34 |
| discrepant | weeks of gestation at delivery | 0.01 | 0.08 | 1.01 | 0.86 | 1.18 | 0.93 |
| discrepant | caregiver felt difficulty at age three | 0.00 | 0.08 | 1.00 | 0.85 | 1.17 | 0.98 |
| discrepant | caregiver psychiatric diagnosis | 0.73 | 0.24 | 2.08 | 1.31 | 3.31 | 0.0019 |
| discrepant | caregiver psychological distress | 0.09 | 0.09 | 1.10 | 0.93 | 1.30 | 0.27 |
| discrepant | household_income | -0.10 | 0.09 | 0.91 | 0.76 | 1.08 | 0.27 |
| discrepant | caregiver scolds child loudly | -0.01 | 0.09 | 0.99 | 0.83 | 1.18 | 0.92 |
| discrepant | caregiver slaps child as part of discipline | -0.01 | 0.09 | 0.99 | 0.83 | 1.19 | 0.95 |
| discrepant | Caregiver feels too little time spent with child | 0.02 | 0.08 | 1.02 | 0.88 | 1.19 | 0.75 |
| discrepant | Caregiver talks with child often | -0.11 | 0.08 | 0.89 | 0.76 | 1.05 | 0.17 |
| discrepant | Caregiver feels she/he can count on partner | 0.00 | 0.09 | 1.00 | 0.84 | 1.20 | 0.99 |
| discrepant | Child does not want to be like father | -0.12 | 0.09 | 0.89 | 0.74 | 1.07 | 0.21 |
| discrepant | Child dissatisfaction with family | 0.06 | 0.08 | 1.06 | 0.90 | 1.25 | 0.46 |
| discrepant | paternal education | 0.00 | 0.09 | 1.00 | 0.84 | 1.20 | 0.96 |
| discrepant | maternal education | 0.07 | 0.09 | 1.07 | 0.91 | 1.27 | 0.42 |
| discrepant | maternal low age | 0.32 | 0.37 | 1.38 | 0.67 | 2.84 | 0.38 |
| discrepant | paternal low age | -0.59 | 0.66 | 0.55 | 0.15 | 2.01 | 0.37 |
| discrepant | Caregiver tells they love child | 0.18 | 0.10 | 1.20 | 0.98 | 1.46 | 0.072 |
| discrepant | Caregiver praises child | -0.02 | 0.10 | 0.98 | 0.81 | 1.19 | 0.84 |
| discrepant | Discipline policy consistency between caregivers | 0.07 | 0.09 | 1.07 | 0.90 | 1.27 | 0.44 |
| discrepant | Caregiver thinks they have good relationship with child | -0.07 | 0.07 | 0.93 | 0.81 | 1.08 | 0.35 |
| discrepant | Caregiver arranges for child to never fail | 0.00 | 0.08 | 1.00 | 0.86 | 1.17 | 0.97 |
| discrepant | Caregiver consistency in discipline | -0.05 | 0.08 | 0.95 | 0.81 | 1.12 | 0.56 |
| discrepant | Partner consistency in discipline | -0.07 | 0.09 | 0.93 | 0.78 | 1.11 | 0.45 |
| discrepant | Child does not want to be like mother | -0.01 | 0.09 | 0.99 | 0.82 | 1.19 | 0.89 |
| severe | (Intercept) | -4.36 | 0.37 | 0.01 | 0.01 | 0.03 | <0.0001 |
| severe | female sex | -0.41 | 0.30 | 0.66 | 0.37 | 1.18 | 0.17 |
| severe | autistic traits | 0.80 | 0.12 | 2.23 | 1.78 | 2.79 | <0.0001 |
| severe | bullying perpetration | -0.87 | 0.38 | 0.42 | 0.20 | 0.88 | 0.021 |
| severe | bullying victimization | 1.26 | 0.27 | 3.52 | 2.07 | 5.98 | <0.0001 |
| severe | exercise habits | -0.24 | 0.12 | 0.79 | 0.63 | 0.99 | 0.043 |
| severe | help-seeking intention | 0.50 | 0.31 | 1.64 | 0.89 | 3.03 | 0.11 |
| severe | smoking during pregnancy | 1.04 | 0.46 | 2.82 | 1.15 | 6.90 | 0.024 |
| severe | weeks of gestation at delivery | -0.17 | 0.09 | 0.84 | 0.71 | 0.99 | 0.044 |
| severe | caregiver felt difficulty at age three | 0.26 | 0.11 | 1.30 | 1.04 | 1.62 | 0.019 |
| severe | caregiver psychiatric diagnosis | 0.13 | 0.37 | 1.14 | 0.55 | 2.36 | 0.73 |
| severe | caregiver psychological distress | 0.63 | 0.10 | 1.88 | 1.54 | 2.31 | <0.0001 |
| severe | household_income | -0.13 | 0.15 | 0.88 | 0.66 | 1.17 | 0.38 |
| severe | caregiver scolds child loudly | 0.43 | 0.15 | 1.53 | 1.14 | 2.05 | 0.0044 |
| severe | caregiver slaps child as part of discipline | 0.27 | 0.13 | 1.31 | 1.02 | 1.68 | 0.036 |
| severe | Caregiver feels too little time spent with child | -0.05 | 0.12 | 0.96 | 0.76 | 1.20 | 0.70 |
| severe | Caregiver talks with child often | 0.10 | 0.14 | 1.10 | 0.84 | 1.44 | 0.48 |
| severe | Caregiver feels she/he can count on partner | -0.01 | 0.14 | 0.99 | 0.75 | 1.30 | 0.94 |
| severe | Child does not want to be like father | 0.31 | 0.14 | 1.36 | 1.04 | 1.79 | 0.025 |
| severe | Child dissatisfaction with family | 0.46 | 0.11 | 1.58 | 1.26 | 1.97 | <0.0001 |
| severe | paternal education | 0.18 | 0.15 | 1.20 | 0.89 | 1.61 | 0.23 |
| severe | maternal education | -0.12 | 0.14 | 0.88 | 0.67 | 1.16 | 0.38 |
| severe | maternal low age | 0.61 | 0.53 | 1.83 | 0.64 | 5.21 | 0.26 |
| severe | paternal low age | -0.15 | 0.76 | 0.86 | 0.19 | 3.86 | 0.85 |
| severe | Caregiver tells they love child | 0.16 | 0.17 | 1.18 | 0.85 | 1.63 | 0.32 |
| severe | Caregiver praises child | -0.12 | 0.17 | 0.89 | 0.64 | 1.23 | 0.47 |
| severe | Discipline policy consistency between caregivers | -0.05 | 0.14 | 0.95 | 0.73 | 1.24 | 0.71 |
| severe | Caregiver thinks they have good relationship with child | -0.16 | 0.13 | 0.85 | 0.65 | 1.10 | 0.22 |
| severe | Caregiver arranges for child to never fail | 0.08 | 0.13 | 1.08 | 0.84 | 1.39 | 0.56 |
| severe | Caregiver consistency in discipline | 0.01 | 0.12 | 1.01 | 0.80 | 1.27 | 0.95 |
| severe | Partner consistency in discipline | -0.14 | 0.14 | 0.87 | 0.66 | 1.15 | 0.33 |
| severe | Child does not want to be like mother | -0.21 | 0.14 | 0.81 | 0.61 | 1.07 | 0.14 |

# eFigure 1: Flowchart of participant recruitment

Abbreviations: T-EAS, the baseline survey named Tokyo Early Adolescence Survey; TTC, the Tokyo Teen Cohort study.

# eFigure 2: Prediction strength for each number of clusters

Abbreviation: k, the number of clusters

# eFigure 3: Average trajectories of five cluster with unified y-scales for problems rated by both the adolescent and the caregiver


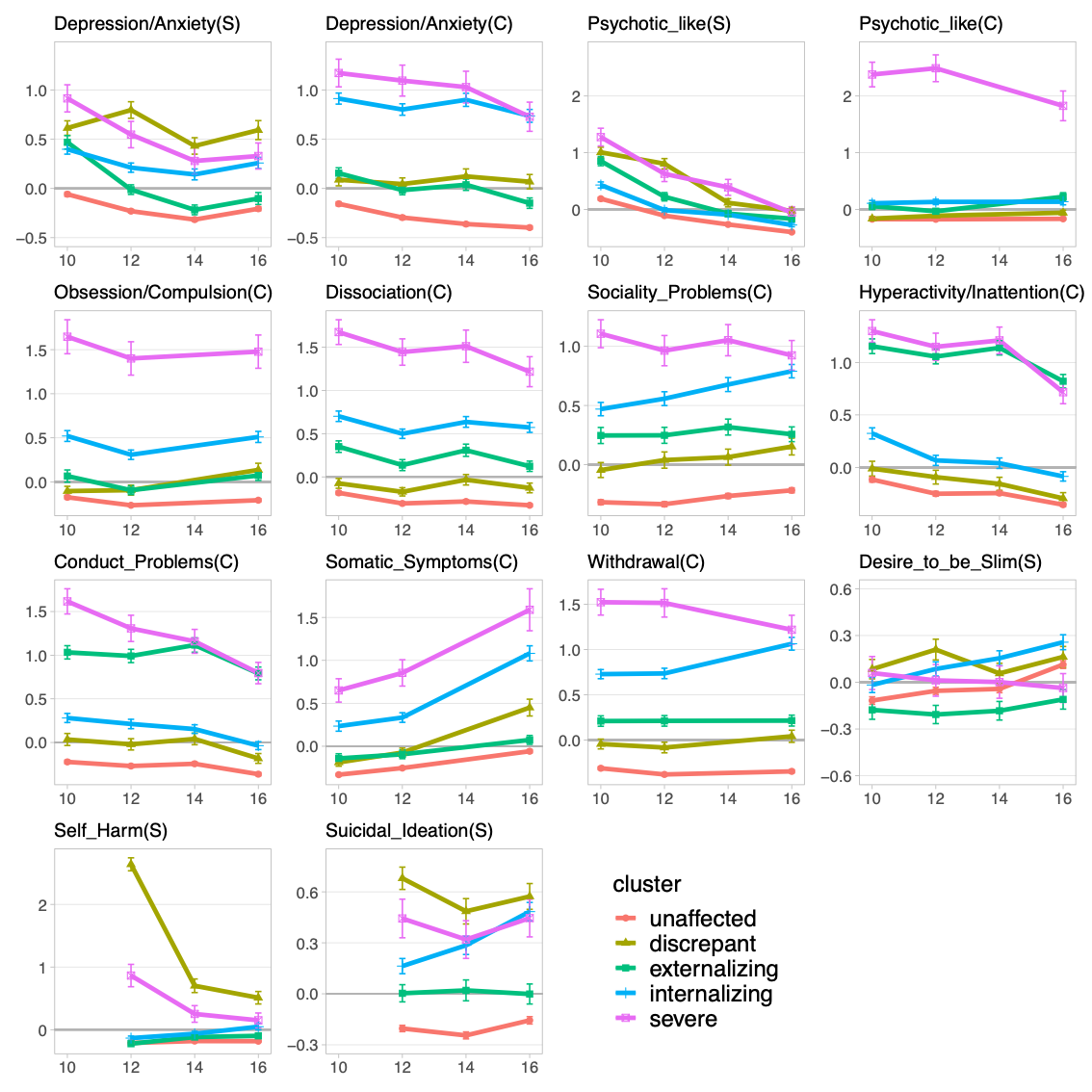


“Depression/Anxiety(S)” should be read as “Depression(S)”.

# eFigure 4: Crude results of univariable multinomial logistic regression at age 10


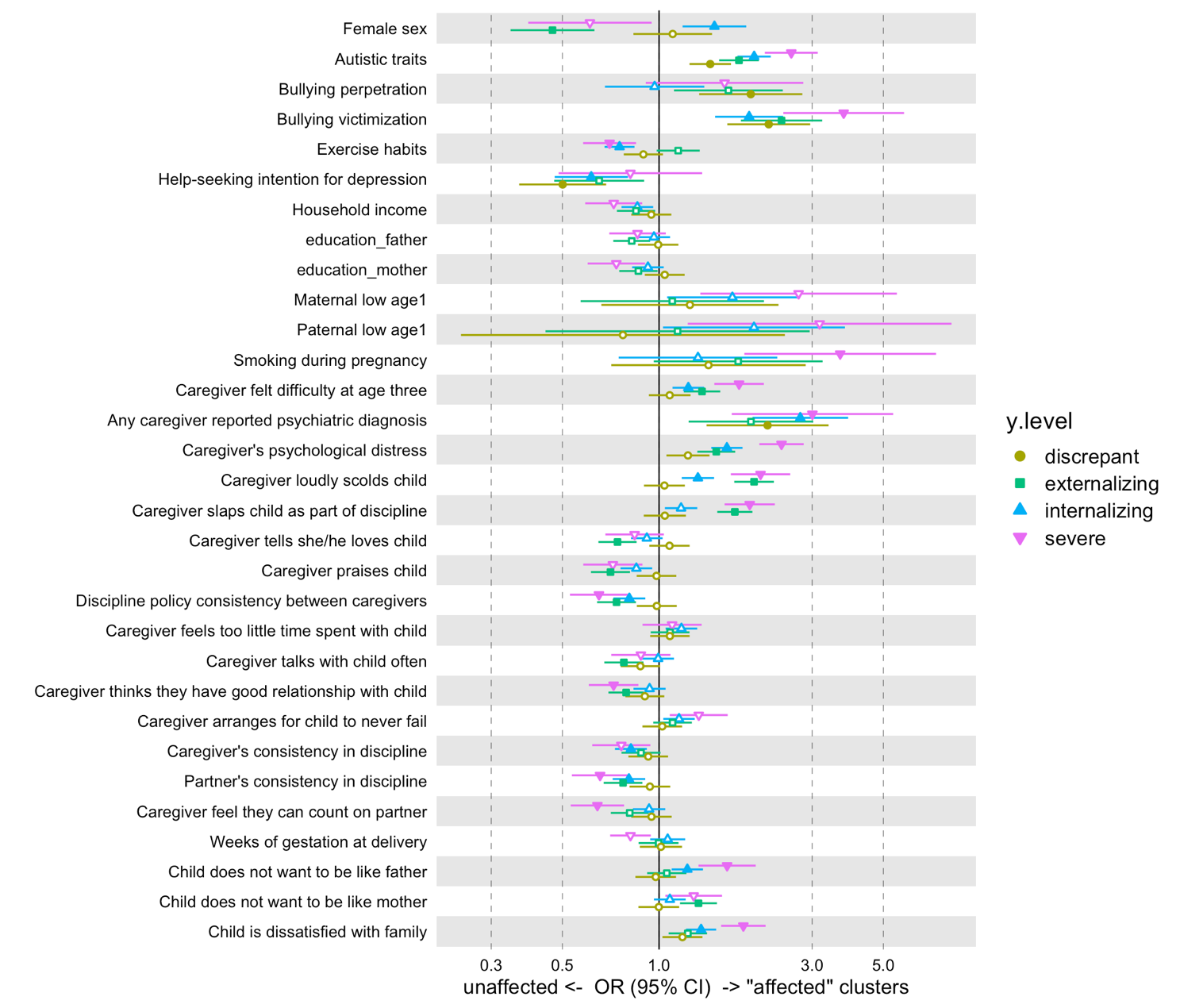


The bilateral significance level was set to 0.0016 using Bonferroni correction to adjust for multiple testing. Filled markers represent significant OR estimates, while blank markers indicate non-significant estimates.

# eFigure 5: Result of clustering all the participants in T1 as sensitivity analysis (n = 3171)

Cluster numbers correspond to the following cluster names: 0, unaffected; 1, internalizing; 2, externalizing; 3, discrepant; 4, severe. “Depression/Anxiety(S)” should be read as “Depression(S)”.

# eFigure 6: Result of clustering without MissForest imputation as sensitivity analysis (n = 2344)

Cluster numbers correspond to the following cluster names: 0, externalizing; 1, unaffected; 2, internalizing; 3, severe; 4, discrepant. “Depression/Anxiety(S)” should be read as “Depression(S)”.

# eFigure 7: Result of clustering using 0/1 binary input for self-harm as sensitivity analysis (n = 2344)

Cluster numbers correspond to the following cluster names: 0, unaffected; 1, severe; 2, externalizing; 3, internalizing; 4, discrepant. “Depression/Anxiety(S)” should be read as “Depression(S)”.

# eFigure 8: Result of multinomial logistic regression with complete antecedent data as sensitivity analysis (n = 1530)


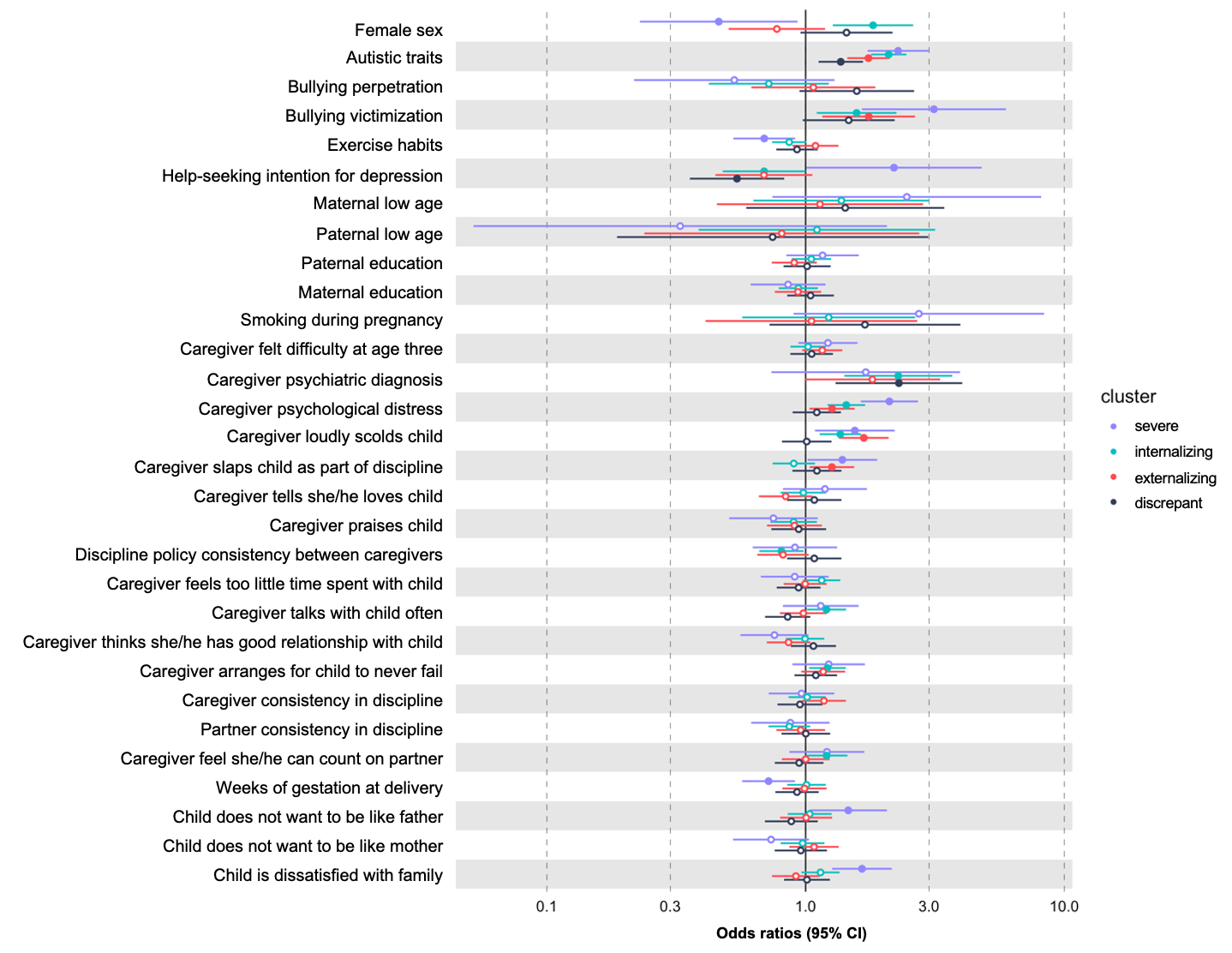


# eFigure 9: Crude results of univariable multinomial logistic regression at ages 12, 14, or 16

**Age 16**

**Age 14**

**Age 12**

Caregiver’s psychological distress was measured using total scores of the General Health Questionnaire-28 (GHQ28)[(Goldberg and Hillier 1979)](https://sciwheel.com/work/citation?ids=1836452&pre=&suf=&sa=0&dbf=0) at T2–T4 instead of K6 at T1. The number of datasets was set to 30 in multiple imputation with chained equations.

# eReferences

[1     Silva TBF, Osório FL, Loureiro SR. SDQ: discriminative validity and diagnostic potential. *Front Psychol* 2015; **6**: 811.](https://sciwheel.com/work/bibliography/5392629)

[2     Achenbach TM. Manual for Child Behavior Checklist 4-18, 1991 Profile. 1991.](https://sciwheel.com/work/bibliography/14334512)

[3     Lengua LJ, Sadowski CA, Friedrich WN, Fisher J. Rationally and empirically derived dimensions of children’s symptomatology: expert ratings and confirmatory factor analyses of the CBCL. *J Consult Clin Psychol* 2001; **69**: 683–98.](https://sciwheel.com/work/bibliography/13432042)

[4     Salcedo S, Rizvi SH, Freeman LK, Youngstrom JK, Findling RL, Youngstrom EA. Diagnostic efficiency of the CBCL thought problems and DSM-oriented psychotic symptoms scales for pediatric psychotic symptoms. *Eur Child Adolesc Psychiatry* 2018; **27**: 1491–8.](https://sciwheel.com/work/bibliography/13432041)

[5     Ivarsson T, Larsson B. The Obsessive-Compulsive Symptom (OCS) scale of the Child Behavior Checklist: a comparison between Swedish children with Obsessive-Compulsive Disorder from a specialized unit, regular outpatients and a school sample. *J Anxiety Disord* 2008; **22**: 1172–9.](https://sciwheel.com/work/bibliography/13432056)

[6     Milot T, Plamondon A, Ethier LS, Lemelin J-P, St-Laurent D, Rousseau M. Validity of CBCL-derived PTSD and dissociation scales: further evidence in a sample of neglected children and adolescents. *Child Maltreat* 2013; **18**: 122–8.](https://sciwheel.com/work/bibliography/7505752)

[7     Sim L, Friedrich WN, Davies WH, Trentham B, Lengua L, Pithers W. The Child Behavior Checklist as an indicator of posttraumatic stress disorder and dissociation in normative, psychiatric, and sexually abused children. *J Trauma Stress* 2005; **18**: 697–705.](https://sciwheel.com/work/bibliography/3677659)

[8     Messer SC, Angold A, Costello J. Development of a short questionnaire for use in epidemiological studies of depression in children and adolescents. *Int J Methods Psychiatr Res* 1995; **5**: 237–49.](https://sciwheel.com/work/bibliography/14334519)

[9     Costello EJ, Edelbrock CS, Costello AJ. Validity of the NIMH Diagnostic Interview Schedule for Children: a comparison between psychiatric and pediatric referrals. *J Abnorm Child Psychol* 1985; **13**: 579–95.](https://sciwheel.com/work/bibliography/14493676)

[10    Endo K, Yamasaki S, Nakanishi M, *et al.* Psychotic experiences predict subsequent loneliness among adolescents: A population-based birth cohort study. *Schizophr Res* 2022; **239**: 123–7.](https://sciwheel.com/work/bibliography/14497662)

[11    Sugimoto N, Nishida A, Ando S, *et al.* Use of social networking sites and desire for slimness among 10-year-old girls and boys: A population-based birth cohort study. *Int J Eat Disord* 2020; **53**: 288–95.](https://sciwheel.com/work/bibliography/12593272)

[12    Tanaka R, Ando S, Kiyono T, *et al.* The longitudinal relationship between dissociative symptoms and self-harm in adolescents: a population-based cohort study. *Eur Child Adolesc Psychiatry* 2023; published online March 8. DOI:10.1007/s00787-023-02183-y.](https://sciwheel.com/work/bibliography/14608021)

[13    Ando S, Nishida A, Usami S, *et al.* Help-seeking intention for depression in early adolescents: Associated factors and sex differences. *J Affect Disord* 2018; **238**: 359–65.](https://sciwheel.com/work/bibliography/12593254)

[14    de Jong J, Emon MA, Wu P, *et al.* Deep learning for clustering of multivariate clinical patient trajectories with missing values. *Gigascience* 2019; **8**. DOI:10.1093/gigascience/giz134.](https://sciwheel.com/work/bibliography/9532154)

[15    Weller BE, Bowen NK, Faubert SJ. Latent class analysis: a guide to best practice. *Journal of Black Psychology* 2020; **46**: 287–311.](https://sciwheel.com/work/bibliography/11597600)

[16    Kurita H, Koyama T, Osada H. Autism-Spectrum Quotient-Japanese version and its short forms for screening normally intelligent persons with pervasive developmental disorders. *Psychiatry Clin Neurosci* 2005; **59**: 490–6.](https://sciwheel.com/work/bibliography/14402415)

[17    Baron-Cohen S, Wheelwright S, Skinner R, Martin J, Clubley E. The autism-spectrum quotient (AQ): evidence from Asperger syndrome/high-functioning autism, males and females, scientists and mathematicians. *J Autism Dev Disord* 2001; **31**: 5–17.](https://sciwheel.com/work/bibliography/876072)

[18    Yamasaki S, Ando S, Richards M, *et al.* Maternal diabetes in early pregnancy, and psychotic experiences and depressive symptoms in 10-year-old offspring: A population-based birth cohort study. *Schizophr Res* 2019; **206**: 52–7.](https://sciwheel.com/work/bibliography/12157963)

[19    Kessler RC, Andrews G, Colpe LJ, *et al.* Short screening scales to monitor population prevalences and trends in non-specific psychological distress. *Psychol Med* 2002; **32**: 959–76.](https://sciwheel.com/work/bibliography/3609491)

[20    Matsuoka K, Uno M, Kasai K, Koyama K, Kim Y. Estimation of premorbid IQ in individuals with Alzheimer’s disease using Japanese ideographic script (Kanji) compound words: Japanese version of National Adult Reading Test. *Psychiatry Clin Neurosci* 2006; **60**: 332–9.](https://sciwheel.com/work/bibliography/14402419)
